# Discovering, Characterizing, and Applying Acyl Homoserine Lactone-Quenching Enzymes to Mitigate Microbe-Associated Problems Under Saline Conditions

**DOI:** 10.3389/fmicb.2019.00823

**Published:** 2019-04-17

**Authors:** Tian-Nyu Wang, Qing-Tian Guan, Arnab Pain, Anna H. Kaksonen, Pei-Ying Hong

**Affiliations:** ^1^Water Desalination and Reuse Center, Biological and Environmental Science and Engineering Division, King Abdullah University of Science and Technology, Thuwal, Saudi Arabia; ^2^Pathogen Genomics Laboratory, Division of Biological and Environmental Science and Engineering, King Abdullah University of Science and Technology, Thuwal, Saudi Arabia; ^3^CSIRO Land and Water, Floreat, WA, Australia

**Keywords:** quorum quenching enzyme, biofouling, salinity, virulence inhibition, AHL, bacterial motility, *Altererythrobacter*

## Abstract

Quorum quenching (QQ) is proposed as a new strategy for mitigating microbe-associated problems (e.g., fouling, biocorrosion). However, most QQ agents reported to date have not been evaluated for their quenching efficacies under conditions representative of seawater desalination plants, cooling towers or marine aquaculture. In this study, bacterial strains were isolated from Saudi Arabian coastal environments and screened for acyl homoserine lactone (AHL)-quenching activities. Five AHL quenching bacterial isolates from the genera *Pseudoalteromonas, Pontibacillus*, and *Altererythrobacter* exhibited high AHL-quenching activity at a salinity level of 58 g/L and a pH of 7.8 at 50°C. This result demonstrates the potential use of these QQ bacteria in mitigating microbe-associated problems under saline and alkaline conditions at high (>37°C) temperatures. Further characterizations of the QQ efficacies revealed two bacterial isolates, namely, *Pseudoalteromonas* sp. L11 and *Altererythrobacter* sp. S1-5, which could possess enzymatic QQ activity. The genome sequences of L11 and S1-5 with a homologous screening against reported AHL quenching genes suggest the existence of four possible QQ coding genes in each strain. Specifically, two novel AHL enzymes, AiiA_S1-5_ and Est_S1-5_ from *Altererythrobacter* sp. S1-5, both contain signal peptides and exhibit QQ activity over a broad range of pH, salinity, and temperature values. In particular, AiiA_S1-5_ demonstrated activity against a wide spectrum of AHL molecules. When tested against three bacterial species, namely, *Aeromonas hydrophila, Pseudomonas aeruginosa*, and *Vibrio alginolyticus*, AiiA_S1-5_ was able to inhibit the motility of all three species under saline conditions. The biofilm formation associated with *P. aeruginosa* was also significantly inhibited by AiiA_S1-5_. AiiA_S1-5_ also reduced the quorum sensing-mediated virulence traits of *A. hydrophila, P. aeruginosa*, and *V. alginolyticus* during the mid and late exponential phases of cell growth. The enzyme did not impose any detrimental effects on cell growth, suggesting a lower potential for the target bacterium to develop resistance over long-term exposure. Overall, this study suggested that some QQ enzymes obtained from the bacteria that inhabit saline environments under high temperatures have potential applications in the mitigation of microbe-associated problems.

## Introduction

Seawater is increasingly being used as an alternative water resource for various purposes to alleviate water demands in water-stressed countries (Elimelech and Phillip, 2011). However, marine microorganisms can attach onto surfaces, propagate and establish biofilm matrices, and frequently, this attachment can result in detrimental consequences. For example, biofilm formation can foul membranes in a seawater desalination plant, in turn reducing the flux (Al-Ahmad et al., 2000). Biofilm formation can also accelerate the biocorrosion of metal pipelines (Enning et al., 2012). Marine pathogens form biofilms on fish and shrimp, which can lead to the mortality and morbidity of these livestock and cause economic losses in marine aquaculture (Mizan et al., 2015). In all instances, biofilm formation increases the capital and operational costs associated with seawater usage.

Conventional antifouling strategies include the use of toxic biocides and coating materials such as tributyltin, copper, chlorine and ozone (Amara et al., 2018). However, there are health, safety and environmental concerns associated with the incessant use of these chemicals. Recently, a quorum quenching (QQ) strategy was proposed as an eco-friendly way to inhibit biofouling by blocking the cell-to-cell communication ability of bacteria (which is also known as quorum sensing, or QS). QS is a cell density-dependent regulatory mechanism used by bacteria to coordinate group behavior in response to QS signals secreted by the cell population. The concentration of QS signals increases as the cell population grows, which, upon reaching a certain threshold value, will trigger the expression of certain genes, including one related to pathogenicity, biofilm formation, spore germination and other functions (Defoirdt, 2018). QQ bacteria and QQ enzymes have been demonstrated to be effective in the membrane fouling mitigation of lab-scale membrane bioreactors (MBRs) used for wastewater treatment (Lee et al., 2018; Oh and Lee, 2018), and in a pilot-scale MBR (Lee et al., 2016).

Several studies have also presented the QQ strategy for mitigating biofouling in marine environments or in seawater desalination plants (Dobretsov et al., 2011; Katebian et al., 2016, 2018). The QQs discovered and applied to date have primarily been restricted to QS inhibitors (e.g., vanillin, cinnamaldehyde, and kojic acid). Nevertheless, several QQ bacteria were tested for biocontrol in marine environments. Tinh et al. (2008) enriched a complex bacterial consortium that exhibited AHL degradation activity, and they introduced this enrichment culture to colonize larval fish guts and demonstrated an improved survival rate from AHL-induced virulence traits by opportunistic bacteria. Torres et al. (2016) further screened for QQ enzymatic activity among 450 bacterial strains that were isolated from a mollusk hatchery, and they identified *Alteromonas stellipolaris* PQQ-42 as a potential AHL-degrading bacterium that increased the survival rate of corals against *Vibrio*. QQ bacteria and enzymes were subsequently shown to be widely distributed in marine sources (Romero et al., 2011, 2012). Marine isolates belonging to the *Erythrobacter, Labrenzia*, and *Bacterioplanes* genera were capable of degrading AHL molecules (Rehman and Leiknes, 2018). However, none of these studies took advantage of the special traits of marine QQ enzyme-secreting bacteria to mitigate marine biofouling. Moreover, these studies did not evaluate if the QQ enzymes were able to mitigate biofouling under the harsh environmental conditions representative of industrial seawater applications. These environmental conditions include high salinity (of up to 58 g/L), high temperatures (of up to 45°C) and an alkaline pH ranging from 7.2 to 8.0 (Scarascia et al., 2016).

To address this knowledge gap, during this study, bacteria isolated from a Saudi coastal habitat and the Red Sea were first screened for their AHL-quenching ability. Five AHL-quenching bacterial isolates from the genera *Pseudoalteromonas, Pontibacillus*, and *Altererythrobacter* were determined to exhibit high AHL quenching at a salinity value of 58 g/L and a pH of 7.8 at 50°C. The genomes of two bacterial isolates were sequenced, and the potential QQ genes from the genomes were screened based on their homologies with reported QQ genes. The possible AHL-quenching genes were verified by expressing the potential QQ genes in recombinant *E. coli* to obtain enzymatically active recombinant proteins for testing. Subsequently, two enzymes (*N*-acyl homoserine lactonase, AiiA and esterase, Est) from *Altererythrobacter* sp. S1-5 were biochemically characterized at different pH, salinity and temperature. The AiiA from *Altererythrobacter* sp. S1-5 was further demonstrated to inhibit marine biofilm formation and the virulence of the opportunistic bacteria *Aeromonas hydrophila, Pseudomonas aeruginosa*, and *Vibrio alginolyticus* under saline conditions. The findings from this study demonstrate the potential feasibility of using QQ bacteria and enzymes to mitigate biofouling under saline conditions. Specifically, it is the first study to demonstrate the presence of AHL-quenching activity in *Altererythrobacter*.

## Materials and Methods

### Sample Collection, Strain Isolation, and Identification

High-salinity artificial seawater (**HSAS**: 51.5 g/L NaCl, 0.74 g/L KCl, 0.99 g/L CaCl_2_, 2.85 g/L MgCl_2_, and 1.92 g/L MgSO_4_; salinity: 58 g/L), high-salinity marine broth medium (**HSMB**: **HSAS** containing 1 g/L yeast extract, 5 g/L peptone), and high-salinity marine agar (**HSMA**: **HSMB** with 15 g/L agar) were used for strain isolation and screening. Marine aquaculture sludge from the Jeddah Fisheries Research Center (JFRC) as well as beach sand and seawater from the Red Sea were collected for the isolation of AHL-quenching bacteria. A 50 mL aliquot of seawater was transferred into a sterile flask. For the sludge and sand, 15 g of each sample was individually poured into separate sterile flasks that contained 50 mL of sterile **HSAS** and 2 g of glass beads (Sigma, St. Louis, MO, United States; diameter: 5 mm). All the inoculated flasks were cultivated at 40°C and 180 rpm for 24 h. Following that incubation, the cultures were serially diluted with **HSAS** and plated onto **HSMA** plates. The agar plates were further incubated at 40°C for 24 h. Colonies showing different morphologies were picked and purified by plate streaking.

The bacterial isolates were identified through partial length 16S rRNA gene sequencing. A single colony of marine isolates was scraped with sterile toothpicks, suspended in 20 μL of sterile water and heated at 95°C for 5 min to achieve cell lysis. One microliter of supernatant was used as the DNA template for polymerase chain reactions (PCRs). A partial 16S rRNA gene was amplified using the universal primers 11F (5′-GTTYGATYCTGGCTCAG-3′) and 1492R (5′-GGYTACCTTGTTACGACTT-3′). The PCR was performed at 95°C for 5 min, followed by 35 cycles of 30 s at 95°C, 30 s at 52°C and 2 min at 72°C, and a final elongation for 5 min at 72°C. The PCR products were purified using a Wizard SV Gel and PCR Clean-Up System (Promega, Madison, WI, United States) and submitted to the KAUST Bioscience core lab for Sanger-based sequencing using the primers 11F, 1492R, 338F (5′-ACTCCTACGGGAGGCAGCAG-3′), and 592R (5′-GWATTACCGCGGCKGCTG-3′). The sequences were paired and searched against the NCBI GenBank database using the BLASTn search algorithm.

### Screening for AHL Quenching Strains From Marine Isolates Under High Salinity

Single colonies of tested strains were inoculated into individual wells of sterile 96-well microtiter plates, and each well contained 200 μL of autoclaved **HSMB** medium. The 96-well plates were incubated at 50°C and 120 rpm for 24 h. After the incubation, the plates were centrifuged at 2,300 × *g* for 10 min. The supernatant in each well was discarded and the cell pellet was washed once with HSAS. The centrifugation process was repeated and the resulting cell pellets were individually resuspended in 200 μL of **HSAS** containing an AHL mixture (0.29 mM C4-HSL, 0.25 mM C6-HSL, 0.22 mM C8-HSL and 0.17 mM 3-oxo-C12-HSL). After incubating at 50°C for 24 h, the culture was centrifuged again at 2300 × *g* for 10 min. Ten microliters of supernatant was collected for residual AHL quantification using an *Agrobacterium tumefaciens* bioassay (Tang et al., 2013). In brief, *A. tumefaciens* NT1 (traR, tra::lacZ749) (Piper et al., 1993) was grown in **AT** minimal medium (10.7 g/L KH_2_PO_4_, 2 g/L (NH4)_2_SO_4_, 78 mg/L MgSO_4_, 13 mg/L CaCl_2_.H_2_O, 5 mg/L FeSO_4_.7H_2_O, 1.4 mg/L MnSO_4_.H_2_O, pH 6.7, and 0.5% filtered glucose, pH = 6.7) at 28°C and 150 rpm overnight. The culture was diluted 1:500 into fresh **AT** minimal medium containing 250 μg/mL 5-bromo-4-chloro-3-indolyl-β-D-galactopyranoside (X-gal, Sigma, St. Louis, MO, United States) to make the *A. tumefaciens* bioassay solution. Ten microliters of each sample used to analyze the residual AHL concentrations were pipetted into individual wells of a sterile 96-well-plate, with each well containing 190 μL of *A. tumefaciens* bioassay solution. After an incubation at 28°C for 12 h, the absorbance of each well at 492 nm and 630 nm was recorded using a SpectraMax 340 PC 384 microplate reader (Molecular Devices LLC, San Jose, CA, United States). The absorbances at 492 nm and 630 nm are a combination of absorption and light scattering by indigo (which is an X-gal degradation product) and biosensor cells (Tang et al., 2013). The residual AHL activity in each sample was expressed as normalized β-galactosidase activity as described in Tang et al. (2013). The quantification was performed in triplicate. The same concentration of AHL mixture in abiotic HSAS solution was incubated under the same conditions described above and used as a negative control when determining the residual AHL activity. The relative AHL quenching efficiency (QE) for each strain was determined using Eq. (1) as follows:

(1) $$QE=\frac{AHLn-AHLs}{AHLn}\times 100\%$$

where AHLn denotes the residual AHL activity of the negative control after the reaction and AHLs denotes the residual AHL activity of bacterial samples after the reaction with potential QQ enzymes. It was previously shown that AHLs can be rather unstable in alkaline and high temperature environments (Yates et al., 2002). Therefore, to eliminate the instability of AHLs caused by abiotic factors after long-term reactions and to denote the AHL activity changes arising from the potential QQ enzymes more accurately, we use AHLn instead of the initial AHL activity spiked into the prior reaction mixture. Strains with a relative AHL QE ≥ 90% were selected for further QQ enzyme screening.

### Localization of AHL Quenching Enzyme in AHL Quenching Isolates

Artificial seawater (**AS**: 29.5 g/L NaCl, 0.74 g/L KCl, 0.99 g/L CaCl_2_, 2.85 g/L MgCl_2_, and 1.92 g/L MgSO_4_; salinity: 36 g/L), marine broth medium (**MB**: **AS** with 1 g/L yeast extract, 5 g/L peptone), and marine agar (**MA**: **MB** with 15 g/L agar) were used for the AHL quenching enzyme experiment. Selected strains were grown in **MB** medium at 37°C overnight. To obtain the potential AHL quenching enzymes present in the intracellular fraction of each bacterial isolate, 5 mL of pure culture was centrifuged at 20,000 × *g* for 10 min, and the cell pellet was suspended in phosphate-buffered saline (PBS) (Fisher Scientific, Hampton, NH, United States). The cells were lysed using a Q500 sonicator (Qsonica, Newtown, CT, United States) at a 45% amplitude for 5 min, with repetitive 15 s pulsating sonication at 45 s intervals. The fraction was centrifuged at 10,000 × *g* for 10 min and filtered through a 0.2 μm cellulose acetate membrane to obtain the lysed cellular extract as filtrate. To obtain the potential AHL-quenching enzymes secreted extracellularly by the bacterial isolates, a cell pellet made from 5 mL of overnight culture was resuspended in 5 mL of **AS**. The cell suspension was incubated at 37°C for 12 h and centrifuged at 20,000 × *g* for 10 min to obtain the supernatant fraction. The cellular extract and supernatant fraction of each strain were fractionated separately using centrifugal filters (MWCO: 10 kDa, Merck, Darmstadt, Germany) prior to the determination of the potential AHL-quenching activity. To demonstrate whether the quenching activity arose from enzymatic activity, the cellular extract and supernatant fraction were also heat-inactivated at 100°C for 30 min and tested for AHL quenching activity. Each sample was mixed with an AHL mixture (0.15 mM C4-HSL, 0.13 mM C6-HSL, 0.11 mM C8-HSL, and 0.08 mM 3-oxo-C12-HSL) and incubated at 37°C for 18 h for the residual AHL determination. PBS buffer and **AS** were used to replace the cell extract and supernatant to create the negative control.

### Genome Sequencing of AHL-Quenching Strains

Two strains (L11 and S1-5) that exhibit potential enzymatic AHL-quenching activity were sequenced for protein-coding genes (CDS) that encode the AHL quenching enzyme. The genomic DNA from L11 and S1-5 was extracted with a QIAGEN Genomic Tips kit (Qiagen, Hilden, Germany). A 20 kb DNA library was prepared for each strain by genomic DNA fragmentation with a Pippin HT size-selection system (Sage Science, Beverly, MA, United States). Single molecular real-time (SMRT) sequencing was performed with a PacBio RS II platform (Pacific Biosciences, Menlo Park, CA, United States). The raw reads were assembled into contigs using a Canu assembler (Koren et al., 2017). The assembled contigs were examined for integrity using dotplots with a GEnome PAir-Rapid Dotter tool (Krumsiek et al., 2007). The assembled contigs were annotated with a RASTtk server (Brettin et al., 2015). The potential AHL quenching ORFs in L11 and S1-5 were screened with NCBI tBLASTn using published AHL quenching genes as reference genes. A structure-based multiple sequence alignment of potential ORFs and published QQ sequences was performed using ESPript (Gouet et al., 1999). Highly homologous ORFs (identify ≥ 25%, coverage ≥ 40%) that share a conserved domain with the reported QQ protein were selected for gene expression evaluation and to test for quenching activity.

### Expression of AHL Quenching Genes in *Escherichia coli*

Eight potential QQ gene sequences from strains L11 and S1-5 were amplified with Q5-hot start polymerase (New England BioLabs, Beverly, MA, United States) using the primers listed in Supplementary Table 1. The PCR amplicons of the QQ genes were either ligated into a pTrcHis A vector (Thermo Fisher, Waltham, MA, United States) by T4 DNA ligase (Thermo Fisher Waltham, MA, United States) or into pET-20b (+) vectors (Merck, Darmstadt, Germany) by Gibson Assembly Master Mix (New England BioLabs, Beverly, MA, United States). Recombinant pTricHis A and pET-20b(+) vectors with correct insertions were transformed into *E. coli* TOP10 (Thermo Fisher, Waltham, MA, United States) and *E. coli* BL21 (DE3) (Merck, Darmstadt, Germany), respectively, for protein expression. The blank vectors pTricHisA and pET-20b(+) were also transformed into *E. coli* TOP10 and *E. coli* BL21 (DE3) and denoted as negative recombinant controls (NC-1 and NC-2). The AiiA gene from *Bacillus mycoides* ATCC 6462 was inserted into pET-20b(+) and expressed in *E. coli* BL21 (DE3) as a positive control sample for QQ activity evaluation.

Overnight cultures of recombinant strains were diluted 1:100 into fresh LB medium supplemented with 100 μg/mL ampicillin (Sigma, St. Louis, MO, United States). The culture was incubated at 30°C and 160 rpm until the cells reached exponential growth (OD_600_ = 0.8). Subsequently, induction was initiated by adding 0.1 mM isopropyl β-D-1-thiogalactopyranoside (IPTG, Thermo Fisher, Waltham, MA, United States) to the culture prior to further incubation at 30°C 120 rpm for 16 h. After the induction, the cell pellet was collected and suspended in PBS buffer. The cell suspension was sonicated, and the supernatant was passed through a 0.2 μm filter to remove any cell debris. The crude enzyme was subjected to SDS-PAGE (sodium dodecyl sulfate - polyacrylamide gel electrophoresis) analysis. Each target protein band was excised from the SDS-PAGE gel and fragmented into short peptides by in-gel digestion treatment with trypsin (Promega, Madison, WI, United States). The digested peptides were subjected to NanoLC MS/MS analysis using a nanopump UltiMate 3000 ultra-high-performance liquid chromatography (UHPLC) binary HPLC system coupled to a Q-Exactive HF mass spectrometer (Thermo Fisher, Waltham, MA, United States). The target proteins were identified by searching against the Swiss-Prot protein sequence database using the Mascot v 2.6 search engine from Matrix Science.

To evaluate the quenching performance of recombinant QQ enzymes, a reaction system containing 10 μM 3-oxo-C12-HSL, 50 μL of crude enzyme, and PBS buffer was incubated at 37°C for 3 h. Ten microliters of reaction mixture was tested for residual AHL activity using the *A. tumefaciens* bioassay. The crude extract of the control strain (NC-1 and NC-2) was used as a negative control. The substrate specificity of the crude enzyme was tested with C4-HSL, C6-HSL, C8-HSL, C10-HSL, C12-HSL, 3-oxo-C6-HSL, 3-oxo-C8-HSL, and 3-oxo-C12-HSL (Sigma, St. Louis, MO, United States). The crude enzyme was mixed with different AHL molecules in PBS buffer, and after the reaction at 37°C, the residual AHL was determined by bioassay.

### Purification of Recombinant QQ Enzyme

The recombinant strain that was verified to possess AHL-quenching activity was induced in 100 mL of LB medium. The crude enzyme was prepared as described above and the recombinant enzyme was purified by affinity chromatography using a 5 mL HisTrap HP column (GE Healthcare, Piscataway, NJ, United States). The column was equilibrated with binding buffer (20 mM sodium phosphate, pH 7.4, 0.5 M NaCl, and 20 mM imidazole). The crude enzyme was diluted with binding buffer and loaded onto the column. The target protein was eluted with 200 mM imidazole; the eluted fractions were pooled and dialyzed in a dialysis tubing (Fisher Scientific, Hampton, NH, United States) with a molecular weight cutoff (MWCO) of 3.5 kDa. The PBS buffer was used as the dialysis medium, and the entire procedure was performed three times to remove the extra imidazole and salt. The solution was then concentrated using a centrifugal filter with a MWCO of 3.5 kDa (Merck, Darmstadt, Germany).

### Biochemical Characterization of Recombinant QQ Enzymes

The optimal pH of the QQ enzyme was tested by incubating a purified enzyme with 5 μM oxo-C12-HSL in 0.1 M citrate-phosphate buffer (pH 3.0–8.0) and 0.1 M glycine-NaOH buffer (pH 9.0–10.0) at 37°C for 1 h. The effect of the salinity on the enzyme activity was evaluated by incubating the enzyme with 5 μM oxo-C12-HSL in PBS buffer (pH 7.4) under different concentrations of sodium chloride (0 M, 0.1 M, 0.5 M, 1 M, 1.5 M, and 2 M) for 1 h. The optimal temperature for the recombinant enzyme activity was determined by mixing the enzyme with 5 μM oxo-C12-HSL under the optimal pH for each enzyme and under different temperatures ranging from 20 to 100°C for 10 min. The same amount of PBS buffer was used instead of the purified enzyme as a negative control for each test. After the reaction, the residual AHL was quantified using a liquid chromatography-tandem mass spectrometry (LC-MS/MS) system. All the reactions were performed in triplicate. For the biochemical characterization of the NAD(P)-dependent enzymes SDR_S1-5_ and SDR_L11_, 1 mM NADPH or NADH (Sigma, St. Louis, MO, United States) was added to each reaction to facilitate the enzymatic activity.

### AHL Quantification by LC-MS/MS

The AHL molecule oxo-C12-HSL was determined with an Agilent 1260 Infinity quaternary liquid chromatograph (Agilent, Santa Clara, CA, United States) equipped with an Agilent Pursuit C18 column (3 μm particle size, 2.0 × 150 mm) and SCIEX Q-TRAP 5500 mass spectrometer (AB SCIEX, Foster City, CA, United States). The separation was performed at room temperature at a mobile phase flow rate of 250 μL/min using the following gradient elution profile: *t* = 0 min, 95% solution A (LC-MS grade water with 0.1% formic acid), 5% solution B (LC-MS grade methanol); *t* = 2 min, 95% solution A, 5% solution B; *t* = 4 min, 50% solution A, 50% solution B; *t* = 6 min, 5% solution A, 95% solution B; *t* = 10 min, 5% solution A, 95% solution B; *t* = 12 min, 95% solution A, 5% solution B; *t* = 18 min, 95% solution A, 5% solution B. The detection was performed in positive ion mode using the parameters listed in Supplementary Table 2.

### Enzymatic Effect on the Motility and Biofilm Formation of Opportunistic Marine Pathogens

The effect of AiiA_S1-5_ on the motility and biofilm formation of *Aeromonas hydrophila, Pseudomonas aeruginosa*, and *Vibrio alginolyticus* (Supplementary Table 3), was investigated. Five microliters of overnight culture for each marine strain was spotted in the center of the plate containing marine broth-soft agar (0.3% and 0.5% agar) prior to the addition of 5 μL of purified AiiA_S1-5_ (0.2 μg). The plate was cultured at 40°C overnight. The slightly higher mesophilic temperature of 40°C was chosen to mimic the temperature experienced by bacteria during industrial processes (e.g., in cooling towers, seawater desalination plants and tropical marine aquaculture). The experiment was repeated six times. The migration of the culture was evaluated by calculating the diameters of the haloes around the spotted area. In addition, the effect of the AiiA_S1-5_ on the biofilm formation was also evaluated. Single colonies of *A. hydrophila, P. aeruginosa*, and *V. alginolyticus* were cultured in **AS** with 2% peptone and incubated at 40°C overnight. Thereafter, 2 × 10^9^ colony forming units (CFU)/mL of cells were mixed with purified AiiA_S1-5_ (0, 5, 10, and 20 μg/mL). The cultures were transferred into 96-well microtiter plates and further incubated at 40°C for 24 h. Thereafter, the supernatants were carefully removed, and the biofilms were stained with 0.2% crystal violet at room temperature for 30 min. Extra dye was removed with **AS** and the stained cells were solubilized with 200 μL of 30% acetic acid (O′Toole, 2011). The absorbance of the solution was measured at 595 nm. PBS buffer was used instead of purified AiiA_S1-5_ as a negative control.

### Transcription of Virulence Genes in Marine Strains in the Presence of Purified AiiA_S1-5_

*A. hydrophila, P. aeruginosa*, and *V. alginolyticus* were grown in marine media at 37°C and 180 rpm overnight. The overnight culture was diluted 1:100 in **MB** medium as mentioned before (salinity: 36 g/L), and 50 μg/mL of purified AiiA_S1-5_ was added to the pathogenic strain culture at the inoculation time. The bacterial cultures were grown at 37°C and 180 rpm. The growth curve of the marine strain with AiiA_S1-5_ or PBS buffer was monitored based on the optical density at 600 nm. The bacterial cultures were sampled at the mid-exponential, late exponential and stationary phases of growth. The samples were used for RNA extraction (RNeasy Mini Kit, Qiagen). cDNA was synthesized with a SuperScript III First-Strand Synthesis Supermix (Thermo Fisher, Waltham, MA, United States). The transcriptional level of each gene (Supplementary Table 4) was calculated using the relative standard curve method. To obtain the standard curve, the PCR amplification product of each gene was first cloned into a PCR Blunt II-TOPO vector (Thermo Fisher, Waltham, MA, United States). Plasmid DNA containing the target gene was serially diluted based on the copy number and used as the standard. Real-time PCR was performed on a 7900HT Fast Real-Time PCR system (Thermo Fisher, Waltham, MA, United States). The reaction system contained 5 μL of Fast SYBR Green master mix (Thermo Fisher, Waltham, MA, United States), 0.2 μL each of the forward and reverse primers (10 μM), and 2 μL of cDNA template (2 ng/μL). The quantification of the target gene was normalized to the reference gene (*rpoB*) in each sample.

### Statistical Analysis

The statistical analysis was performed with Minitab 17. A paired *t*-test was used to determine the statistical significance of the difference. The difference was defined as statistically significant when *P* < 0.05.

## Results

### Bacterial Screening From Marine Sources and the AHL Quenching Test

A total of 51 bacterial isolates that can grow at a high salinity (58 g/L) and a high temperature of 50°C were obtained for QQ screening (Supplementary Table 5). The AHL mixture (0.29 mM C4-HSL, 0.25 mM C6-HSL, 0.22 mM C8-HSL and 0.17 mM 3-oxo-C12-HSL) was used to screen the QQ bacteria under high salinity (58 g/L) and a high temperature (50°C). These AHL quenchers belong to the following three phyla: Firmicutes (*Staphylococcus, Bacillus, Halobacillus, Virgibacillus, Pontibacillus, Aquibacillus*, and *Thalassobacillus*), Bacteroidetes (*Tamlana, Tenacibaculum, Vibrio*, and *Mesoflavibacter*), and Proteobacteria (*Delftia, Bacterioplanes, Altererythrobacter, Devosia, Halomonas*, and *Pseudoalteromonas*). Among the QQ isolates, five showed ≥90% relative AHL QE toward AHL mixtures (Supplementary Table 1 and Supplementary Figure 1). These five QS-quenching bacteria belong to the Pseudomonadaceae (Proteobacteria), Bacillaceae (Firmicutes), and Erythrobacteraceae (Proteobacteria), with three of them belonging to the genus *Altererythrobacter* in the Erythrobacteraceae (Table 1).

**Table 1:** Marine bacteria that showed 90–100% relative AHL quenching efficiency under saline condition and at high temperature.

Strain name	Best matched 16S rRNA gene identification	Accession number for 16S rRNA gene sequences	*E*-value	Similarity percentage
L11	*Pseudoalteromonas* sp. (JQ237129.1)	MK575497	0.0	98%
L12	*Pontibacillus* sp. (MG252492.1)	MK575888	0.0	99%
S1-1	*Altererythrobacter marinus* (MF716636.1)	MK578236	0.0	99%
S1-5	*Altererythrobacter* sp. (KC169804.1)	MK574878	0.0	98%
S1-6	*Altererythrobacter marinus* (NR_116432.1)	MK578235	0.0	99%


### Size Fractionation of the Bacterial Cell Extract and Supernatant Contents to Identify the AHL-Quenching Enzymes

**FIGURE 1:**
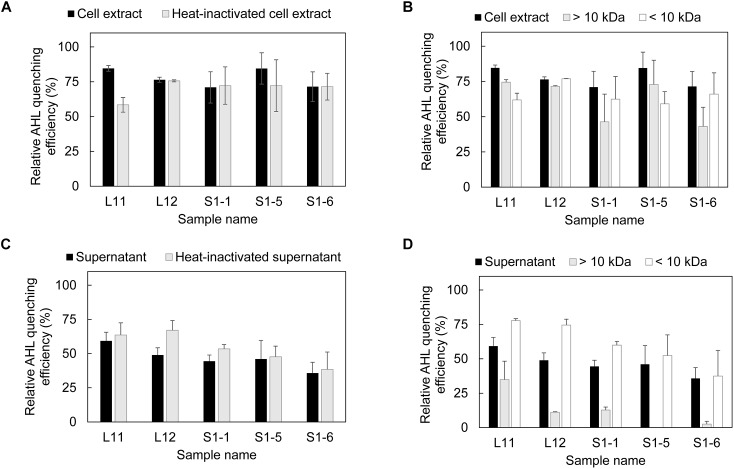
Presence of AHL quenching enzymes in each fraction of AHL quenching bacteria. **(A)** Evaluation of AHL quenching activity in cell extracts and heat-inactivated cell extracts, **(B)** separation of cell extract based on molecular size (10 kDa) and evaluation of the AHL quenching effect in each fraction, **(C)** detection of AHL quenching activity in bacterial supernatant and heat-inactivated supernatant, and **(D)** separation of bacterial supernatant based on the molecular size and evaluation of the AHL quenching effect in each fraction. Each fraction was mixed with AHL mixture (0.15 mM C4-HSL, 0.13 mM C6-HSL, 0.11 mM C8-HSL and 0.08 mM 3-oxo-C12-HSL) and incubated at 37°C for 18 h for residual AHL determination using a biosensor. The relative AHL quenching efficiency of each sample was calculated based on Eq. (1), as stated in the section “Materials and Methods.” PBS buffer and AS were used to replace the cell extract and supernatant to create the negative control. Three biological replicates were performed, and the results were expressed as the means ± standard error.

Both the cell extract and supernatant fraction obtained from the five bacterial isolates showed the ability to inhibit AHL (Figure 1), with the cell extract having a higher AHL inhibition performance than the supernatant (Figure 1A,C). After heat inactivation, the cell extracts of L11 and S1-5 showed decreased AHL inhibition activity, while no decrease in AHL inhibition activity was detected for the other isolates (Figure 1A). This result corresponds with the size fraction experiment for the L11 and S1-5 cell extract (Figure 1B), in which a higher relative AHL QE was observed in the >10 kDa portion. This size fraction was generally found to contain potential QQ enzymes (Czajkowski and Jafra, 2009). For the other isolates, the relative AHL quenching activity was higher in the <10 kDa portion, suggesting the presence of quorum signal inhibitors (QSIs). The supernatant of the isolates maintained the same AHL quenching activity before and after heat inactivation (Figure 1C), and the <10 kDa fractions in the supernatant contributed more to the quenching effect than the larger size fractions (Figure 1D). Both the cell extract and the supernatant of the five strains showed similar QQ performance at 50°C (Supplementary Figures 2A,C) except for the presence of QQ activity found in the >3 kDa fraction of the S1-5 supernatant (Supplementary Figures 2B,D).

### Genomic Sequencing of the Two Bacterial Isolates S1-5 and L11 That Potentially Possess Enzymatic QQ Activity

One closed contig was assembled from S1-5 raw reads, and it had a sequence length of 3.35 Mbp and a GC content of 66.3%. Two contigs were assembled from the L11 raw reads; both contigs were predicted to be closed, with no gap detected (Supplementary Figure 3). Contig 1 has a sequence length of 2.89 Mbp (GC content of 46%) and Contig 2 has a full length of 0.64 Mbp (GC content of 45.2%). The sequencing files were deposited in the European Nucleotide Archive (ENA) under study accession number PRJEB30480. Eight potential QQ enzyme genes from S1-5 and L11 (Table 2) were classified as *N*-acyl homoserine lactonase (AiiA), oxidoreductase (SDR), esterase (Est), hydrolase (Hyd), a penicillin acylase family protein (PvdQ) and a hypothetical protein (HP). These ORFs all shared a conserved motif with the reference AHL-quenching enzymes (Supplementary Figure 4).

**Table 2:** Screening of potential ORFs from strains L11 and S1-5 using the reported AHL quenching enzymes.

Strain name	ORF name	Protein size (kDa)	Signal peptide prediction	Asp + Glu (%)	Hypothetical function	Superfamily	References QQ ORF	Coverage	Identity	*E*-value
S1-5	AiiA_S1-5_	29.6	√	13.2	N-acyl homoserine lactonase (AiiA)	Metallo-hydrolase-like_MBL-fold superfamily	AiiA *Bacillus thuringiensis* (Liu et al., 2008)	84%	26%	2e-24
	SDR_S1-5_	25.3	–	10.2	NAD(P)-dependent oxidoreductase (SDR)	Short-chain dehydrogenase/reductase (SDR) family	BpiB09 soil metagenome (Bijtenhoorn et al., 2011)	95%	41%	1e-45
	Est_S1-5_	31.8	√	9.9	Esterase (Est)	α/β hydrolase family	dlhR *Rhizobium sp.* NGR234 (Krysciak et al., 2011)	45%	36%	4e-07
	Hyd_S1-5_	38.3	–	11.9	α/β hydrolase (Hyd)	α/β hydrolase family	AidH *Ochrobactrum* sp. (Mei et al., 2010)	49%	28%	1E-11
L11	Hyd_L11_	28.8	–	13.3	α/β hydrolase (Hyd)	α/β hydrolase family	Est816 soil metagenome (Fan et al., 2012)	83%	25%	1e-16
	PvdQ_L11_	83.8	–	11.3	Penicillin acylase family protein (PvdQ)	Ntn_hydrolase superfamily	QuiP *P. aeruginosa* PAO1 (Huang et al., 2006)	96%	25%	2e-43
	SDR_L11_	26.8	–	12.0	Oxidoreductase (SDR)	SDR family NAD(P)-dependent oxidoreductase	BpiB09 soil metagenome (Bijtenhoorn et al., 2011)	98%	32%	6e-42
	HP_L11_	36.0	–	10.8	Hypothetical protein (HP)	DUF523 and DUF1722 domain-containing protein	QQ-16d *Pseudoalteromonas* sp. (Weiland-Bräuer et al., 2015)	99%	48%	8e-106


*Potential QQ ORFs were screened by tBLASTn using known QQ enzymes published as references.*

### Verification of Identified QQ Enzymes for AHL Quenching Activity and Specificity

**FIGURE 2:**
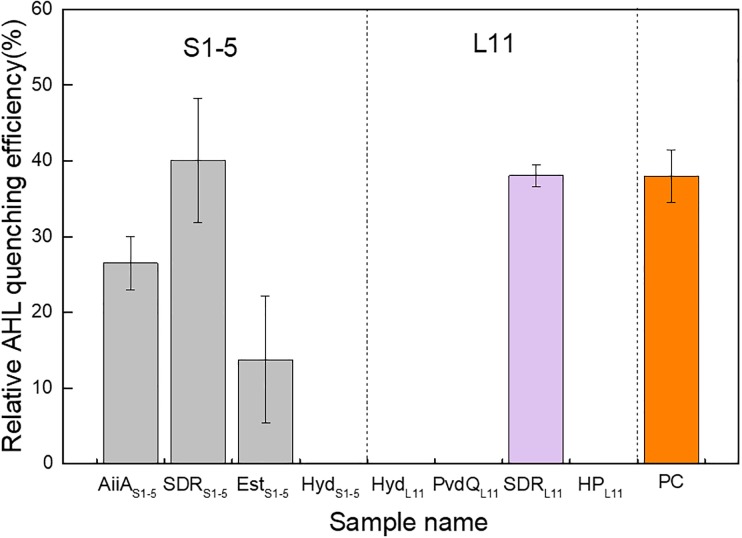
AHL quenching activity comparison of eight potential protein-coding genes expressed in recombinant *Escherichia coli* cells. The relative AHL quenching efficiency of each sample was calculated based on Eq. (1), as stated in the “Materials and Methods” section. The *E. coli* TOP10 carrying vector pTricHisA and *E. coli* BL21 (DE3) carrying pET-20b(+) were used as negative controls. AiiA_S1-5_ (acyl homoserine lactonase), SDR_S1-5_ (oxidoreductase), Est_S1-5_ (esterase), and Hyd_S1-5_ (hydrolase) are possible AHL quenching ORFs from *Altererythrobacter* sp. S1-5; Hyd_L11_ (hydrolase), PvdQ_L11_ (penicillin acylase family protein), SDR_L11_ (oxidoreductase), and H_PL11_ (hypothetical protein) are possible AHL quenching ORFs from *Pseudoalteromonas* sp. L11. PC denotes a positive control, which is obtained by inserting an AiiA gene from *Bacillus mycoides* ATCC 6462 into pET-20b(+) and expressing it in *E. coli* BL21 (DE3). Two independent biological replicates with three technical replicates in each biological replicate were performed for this experiment.

The identified ORFs were expressed in recombinant *Escherichia coli*. The crude enzyme and cell lysate from recombinant *E. coli*, along with the *E. coli* host that contained blank expression vectors (NC-1 and NC-2), were individually mixed with 5 μM 3-oxo-C12-HSL and tested for residual AHL concentration. Four ORFs, namely, AiiA_S1-5_, SDR_S1-5_, Ests_1-5_ and SDR_L11_, were verified to be AHL quenchers. The enzymes showed 26.5%, 40.1%, 13.8%, and 38.0% relative AHL QE compared with the negative controls (Figure 2). The positive control made up of AiiA from *B. mycoides* ATCC 6462 showed a 37.9% relative AHL QE compared with the negative control. The level of activity exhibited by the positive control was comparable to that observed for both SDRs from L11 and S1-5. The AHL specificity test further showed that AiiA_S1-5_ showed catalytic activity toward all the tested AHLs, and Est_S1-5_ can quench all the AHLs except C4-HSL and C6-HSL. Both SDR_S1-5_ and SDR_L11_ can quench all the 3-oxo-AHLs. Moreover, SDR_L11_ showed a quenching effect on C8-HSL (Supplementary Table 6).

### Biochemical Characterization of Recombinant QQ Enzymes

**FIGURE 3:**
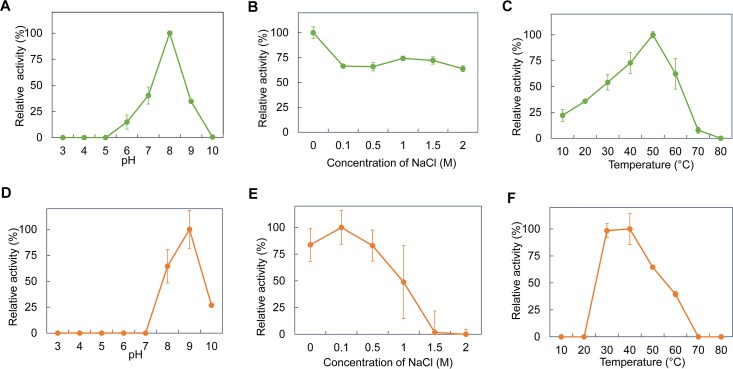
Biochemical properties of purified AiiA_S1-5_ and Est_S1-5._ The relative enzyme activity of AiiA_S1-5_ in/at different **(A)** pH buffers (0.1 M citrate-phosphate buffer, pH 3.0–8.0; 0.1 M glycine-NaOH buffer, pH 9.0–10.0), **(B)** NaCl concentrations (0, 0.1, 0.5, 1, and 2 M), and **(C)** temperatures of 10–80°C. The relative enzyme activity of Est_S1-5_ in/at different **(D)** pH buffers, **(E)** NaCl concentrations, and **(F)** temperatures. The relative enzyme activity of the purified enzymes was normalized using the activity of the purified enzymes at the optimal pH, salinity, and temperature.

AiiA_S1-5_ and Est_S1-5_, SDR_S1-5_ and SDR_L11_ were further induced and purified for biochemical characterization at varying pH values, salt concentrations and temperatures. However, during the course of this 1-month experiment, the purified SDR_S1-5_ and SDR_L11_ did not exhibit good stability while they were in storage compared to AiiA_S1-5_ and Est_S1-5._ The purified SDR_S1-5_ and SDR_L11_ consistently did not show any AHL-quenching activity after storage despite the addition of NADH or NADPH (data not shown). Hence, the biochemical characterization of only AiiA_S1-5_ and Est_S1-5_ was performed using 3-oxo-C12-HSL as the substrate. AiiA_S1-5_ exhibited >25% relative activity against 3-oxo-C12-HSL between pH 7 and 9, with the highest activity observed at pH 8.0 (Figure 3A). AiiA_S1-5_ showed the maximum relative activity in PBS buffer, but it was still able to retain more than 63% of its activity at 0.1–2 M NaCl concentrations (Figure 3B). AiiA_S1-5_ showed the highest activity at 0.5 M KCl, and it maintained >70% activity at 0–2 M KCl (Supplementary Figure 5A). The relative activity of AiiA_S1-5_ increased from 10 to 50°C (the optimal temperature range) before decreasing rapidly to 7.8%, when the temperature was further increased to 70°C. The enzyme was fully inactivated at 80°C (Figure 3C). Est_S1-5_ exhibited the highest relative activity at pH 9.0, and it retained 26.9% of its activity at pH 10.0 (Figure 3D). Est_S1-5_ showed optimal activity at 0.1 M NaCl, and it retained more than 83% of its activity at NaCl concentrations between 0 and 0.5 M (Figure 3E). The relative activity of Est_S1-5_ decreased with the increasing KCl concentration (Supplementary Figure 5B). The enzyme showed the highest activity at both 30 and 40°C, and it was still able to maintain >30% of its relative activity at high temperatures of 50 and 60°C (Figure 3F).

### Effect of Purified AiiA_S1-5_ on Marine Bacteria

**FIGURE 4:**
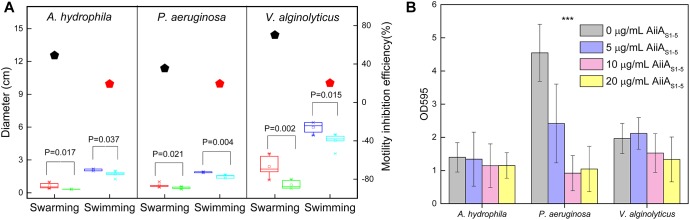
Effect of purified AiiA_S1-5_ on the motility and biofilm formation of marine strains. **(A)** Swarming and swimming of *A. hydrophila, P. aeruginosa*, and *V. alginolyticus* on soft MB agar (MB medium with 0.3% and 0.5% agar) in the presence of PBS or 0.2 μg of purified AiiA_S1-5_ at 40°C for 12 h. The red and green boxes represent the swarming diameters of the marine strains in the presence of PBS and AiiA_S1-5._ The blue and magenta boxes represent the swimming diameters of the marine strains in the presence of PBS and AiiA_S1-5._ The inhibition efficiency of AiiA_S1-5_ on the swarming and swimming of the strains under saline conditions is shown in the right *y*-axis, and the inhibition efficiency was calculated using the following equation: (*P*m – *E*m)/*P*m × 100%, where *P*m and *E*m represent the motility halos of each strain in centimeters in the presence of PBS buffer and the purified enzyme AiiA_S1-5_. **(B)** The effect of 0, 5, 10, and 20 μg/mL of purified AiiA_S1-5_ on the biofilm formation of *A. hydrophila, P. aeruginosa*, and *V. alginolyticus.* The biofilm formation of the marine bacteria was developed on artificial seawater (**AS**) with 2% peptone extracted from casein in 96-well polystyrene plates at 40°C for 24 h. ^∗^*P* < 0.05; ^∗∗^*P* < 0.01; ^∗∗∗^*P* < 0.001.

Purified AiiA_S1-5_ was selected for further testing due to its stability, broad substrate specificity and high enzymatic activity in comparison to Est_S1-5_. The swarming and swimming of *A. hydrophila, P. aeruginosa*, and *V. alginolyticus* all decreased in the presence of AiiA_S1-5_ compared with the negative control (Figure 4A and Supplementary Figure 6). Purified AiiA_S1-5_ reduced swarming more significantly than swimming. There were observed reductions of 48.9%, 35.4%, and 70.2% in swarming in *A. hydrophila, P. aeruginosa*, and *V. alginolyticus*, respectively, compared to their respective controls, which were not exposed to the enzyme. Purified AiiA_S1-5_ reduced swimming consistently by only 20% for all three strains compared to the controls (Figure 4A). An inhibitory effect on biofilm formation was observed in *P. aeruginosa*, in which AiiA_S1-5_ significantly inhibited biofilm formation by 46.9%, 79.8%, and 77.0% at 5 μg/mL, 10 μg/mL, and 20 μg/mL of enzyme (*p* < 0.001 compared to the control). The *P. aeruginosa* biofilm inhibitory effects at 10 μg/mL and 20 μg/mL AiiA_S1-5_ were similar (*p* > 0.05). By contrast, a 32.2% biofilm inhibition in *V. alginolyticus* was only observed when 20 μg/mL of AiiA_S1-5_ was applied (*p* = 0.04). AiiA_S1-5_ has no effect on *A. hydrophila* at all the tested levels of the purified enzyme (*p*-value > 0.05 compared to the control, Figure 4B).

### Transcription of the Virulence Gene in Marine Strains in the Presence of AiiA_S1-5_

**FIGURE 5:**
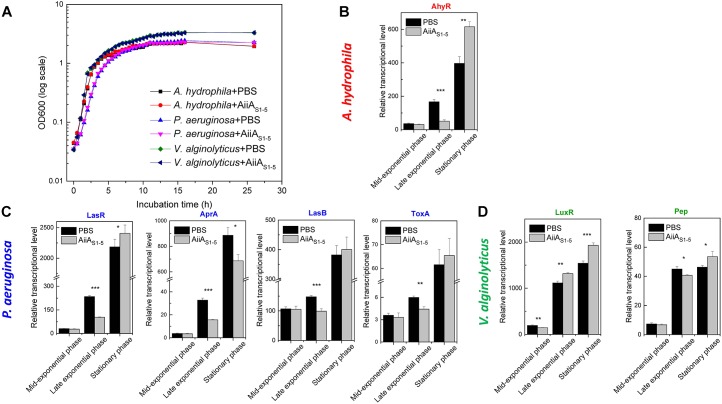
RT-qPCR analysis on the relative expression of selected virulence-associated genes in *A. hydrophila, P. aeruginosa*, and *V. alginolyticus* on MB medium in the presence of PBS (negative control) or AiiA_S1-5_ at 37°C. **(A)** Growth curves of *A. hydrophila, P. aeruginosa* and *V. alginolyticus* in the presence of PBS or AiiA_S1-5_. The relative transcription levels of genes from **(B)** *A. hydrophila*, **(C)** *P. aeruginosa*, and **(D)** *V. alginolyticus* in the presence of PBS and purified AiiA_S1-5_. The transcriptional levels of pathogenicity-related genes at different growth stages were normalized to the reference gene *rpoB*. Information on these genes can be found in Supplementary Table 4. The results are presented as the means ± standard deviation (*n* = 3). ^∗^*P* < 0.05; ^∗∗^*P* < 0.01; ^∗∗∗^*P* < 0.001.

The growth curve of three marine bacteria (*A. hydrophila, P. aeruginosa*, and *V. alginolyticus*) showed no difference in the presence or absence of purified AiiA_S1-5_ (Figure 5A). At mid-exponential phase, the transcription levels of the QS-mediated global regulators AhyR and LasR for *A. hydrophila* (Figure 5B) and *P. aeruginosa* (Figure 5C) were not significantly inhibited by AiiA_S1-5_ (*p* > 0.05). However, AiiA_S1-5_ significantly inhibited AhyR and LasR expression by 56.1% (*p* < 0.0001) and 69.0% (*p* = 0.0003) compared to the sample treated with PBS (control) during the late exponential phase. However, the inhibitory effects on both regulators were no longer observed during the stationary phase. The downstream virulence traits AprA, LasB, and ToxA were regulated by the global regulator *LsrR*, and therefore, they exhibited a similar trend as that observed for LasR in *P. aeruginosa* (Figure 5C). AprA is an exception to this trend, in which an inhibition of 22.5% was still observed at the stationary phase (Figure 5C, *p* = 0.01). By contrast, for *V. alginolyticus*, an inhibitory effect was only observed on LuxR transcription at the mid-exponential phase and not at the late exponential and stationary phases. This result likewise detrimentally affected the expression of the *Pep* virulence gene in *V. alginolyticus* only at the mid-exponential phase (*p* = 0.02) (Figure 5D).

## Discussion

Microbe-associated problems in the marine environment (e.g., seawater desalination plants, seawater cooling towers, and aquaculture) are conventionally mitigated by means of biocides or antibiotics that cause health, safety and environmental concerns. QS inhibitors are increasingly being explored as alternative agents to mitigate these problems (Dobretsov et al., 2011). However, research has shown that bacteria can develop resistance to QS inhibitors (Maeda et al., 2012; García-Contreras et al., 2013), rendering the treatment ineffective over the long term. In comparison, QQ enzymes that degrade extracellular QS signals are viewed as imposing less of a burden on cellular metabolism (Fetzner, 2015), and hence, they minimize the development of resistance toward these greener inhibitory agents. In most instances, QQ enzymes or bacteria with enzymatic activity from marine sources (Huang et al., 2012; Mayer et al., 2015; Tang et al., 2015; Liu et al., 2017; Rehman and Leiknes, 2018) were demonstrated for their efficiencies to quench QS in only minimal or nutrient medium, which deviate significantly from conditions in marine industrial systems such as seawater desalination plants (salinity: 46,400 ppm, temperature: 22–33°C, and pH 8.1–8.3) (Khawaji et al., 2007) and cooling towers (e.g., salinity: >35,000 ppm, and temperature 32–48°C) (Al-Bloushi et al., 2018).

In this study, five bacterial isolates belonging to *Pseudoalteromonas, Pontibacillus*, and *Altererythrobacter* demonstrated high QQ activity at a salinity of 58 g/L, 50°C, and a pH of 7.8. To the best of our knowledge, this is the first report on the existence of QQ activity in *Pontibacillus and Altererythrobacter*. The enzymatic QQ activity was primarily discovered in the intracellular fraction of *Pseudoalteromonas* sp. L11 and to a certain extent, it was also found in *Altererythrobacter* sp. S1-5 (Figure 1). So far, many bacteria with either intracellular or extracellular QQ enzymatic activity (Kim et al., 2014; Fetzner, 2015) have been reported, but the extracellular activity is more feasible. For example, Cheong et al. (2013) entrapped the bacteria in a vessel that allows only the extracellular enzymes to pass through and react with AHL in a lab-scale membrane bioreactor used for wastewater treatment. This exclusion eliminated the need for an additional step of lysing the bacterial hosts to retrieve the intracellular enzymes. However, no similar demonstration has been performed in saline environments.

To look for possible QQ enzymes for marine application purposes, the genes identified in L11 and S1-5 that shared homologies with the existing known QQ genes were first expressed in recombinant *E. coli*, purified and characterized further. Using this approach, we found four protein coding genes that shared high homology with the existing QQ genes in *Altererythrobacter* sp. S1-5, but only three demonstrated QQ enzymatic activity when expressed in recombinant *E. coli.* Similarly, we found four homologous QQ ORFs in L11. Both PvdQ_L11_ and HP_L11_ shared 24% and 48% of their identity with the reported pfmA and QQ-16d enzymes from the same genera (Weiland-Bräuer et al., 2015; Liu et al., 2017). However, a further examination of each of the individual purified enzymes from L11 showed that only SDRL_11_ was positive for AHL quenching activity. This may be because the enzyme from the halophilic microorganism was folded incorrectly or maintained poor stability during recombinant expression in its mesophilic host under low-salt conditions (Madern et al., 2000). Among the three ORFs in *Altererythrobacter* S1-5 that showed QQ activity, an N-terminal peptide was predicted for both AiiA_S1-5_ and Est_S1-5_, suggesting the possibility that the extracellular enzymes would be feasible for use in practical applications. Most QQ enzymes reported thus far do not have a signal peptide, except for AiiA in the marine organisms *Muricauda olearia* (Tang et al., 2015) and *Erythrobacter flavus* (Rehman and Leiknes, 2018) and dlhR in *Rhizobium* sp. (Krysciak et al., 2011).

After purification, only the AiiA_S1-5_ and Est_S1-5_ belonging to the AHL lactonase showed a capacity to degrade AHL. To date, the reported AHL lactonases primarily belong to the metallo-β-lactamase superfamily, the phosphotriesterase family and the α/β hydrolase family. The phosphotriesterase family from the archaeon *Sulfolobus solfataricus* and the crenarchaeon *Vulcanisaeta moutnovskia* (Hiblot et al., 2015) are the most thermophilic QQ enzyme group discovered so far, and they display activity at temperatures of 85–95°C (Merone et al., 2005; Hiblot et al., 2012). The Est816 from the α/β hydrolase family screened from the Turban Basin metagenome can degrade AHL at an optimal temperature of 60°C. Compared to the other two families of AHL lactonases, the metallo-β-lactamase superfamily has a mild optimal catalytic temperature range from 30 to 50°C (Seo et al., 2011; Cao et al., 2012; Sakr et al., 2013; Pedroza et al., 2014; Vinoj et al., 2014; Tang et al., 2015). The AiiT from the thermophilic bacteria *Thermaerobacter marianensis* is an exception, in that it exhibited optimal QQ activity at 60–80°C (Morohoshi et al., 2015). Similarly, the purified AiiA_S1-5_ and Est_S1-5_ obtained during this study can degrade AHL at temperatures of 50°C and 30–40°C, respectively.

To assess if these enzymes can work under saline conditions, the activity of the two enzymes was tested in different concentrations of NaCl and KCl. The AiiA_S1-5_ maintained >63% and >70% relative activity in the presence of 0–2 M NaCl and KCl, respectively (Figure 3 and Supplementary Figure 5), which implied high enzyme robustness under saline conditions. Halophilic proteins usually share common physical features, namely, a high percentage of negatively charged amino acids along the surface area, a high number of salt bridges, low hydrophobicity, and a flexible protein structure (Madern et al., 2000; Takahashi et al., 2018). The 3D structural modeling of AiiA_S1-5_ (without a signal sequence) suggested that there was a high percentage of negatively charged amino acids along the surface area (Supplementary Figure 7). Twenty-four salt bridges were predicted from AiiA_S1-5_ (Supplementary Table 7), which is higher than that predicted from the AiiA described in an earlier study (Easwaran et al., 2015). This physical property of AiiA_S1-5_ can perhaps account for its ability to exhibit activity under saline conditions, and it is consistent with that of another AiiA (TSAWB) from *Bacillus* sp. that can tolerate up to a 5% salt solution (Easwaran et al., 2015).

AiiA_S1-5_ outperformed the other enzymes discovered in this study, and it was further chosen to demonstrate its QQ efficacy against *A. hydrophila, P. aeruginosa*, and *V. alginolyticus.* Both *P. aeruginosa* and *A. hydrophila* were previously reported to be commonly associated with fouled membranes in a seawater desalination plant (Hong et al., 2016; Yap et al., 2017; Nagaraj et al., 2018), while *V. alginolyticus* (Bermont-Bouis et al., 2007) and *P. aeruginosa* (Hamzah et al., 2014) were reportedly possibly linked to pipeline biocorrosion by seawater. Furthermore, these three bacterial species constitute the dominant pathogenic bacterial group reported in marine aquaculture (Sindermann, 1984). It was observed that AiiA_S1-5_ did not impose detrimental effects on cell growth, which reiterates its lower possibility of developing resistance against QQ enzymes over long-term usage. Instead, purified AiiA_S1-5_ showed an inhibitory effect against bacterial motility, biofilm formation and virulence transcription in three marine bacteria under saline conditions (salinity: 36 g/L). The biofilm inhibitory effect of AiiA_S1-5_ in *P. aeruginosa* is more significant than that of the other two bacteria. This distinction was likely caused by the substrate affinity of AiiA_S1-5_ toward the different AHLs secreted by the tested strains (Supplementary Table 3). A 10 μg/mL concentration of AiiA_S1-5_ inhibited the biofilm formation of *P. aeruginosa* to ca. 80% under saline conditions. Similarly, purified AHL-lactonase (100 μg/mL) from *Enterobacter aerogenes* VT66 inhibited >70% of the *P. aeruginosa* PAO1 biofilm (Rajesh and Rai, 2015). *Labrenzia* sp. VG12 cells with AHL lactonase activity also reduced the *P. aeruginosa* biofilm by 25% (Rehman and Leiknes, 2018), although the exact enzymatic concentration used here was not made known. These earlier studies, along with that reported here, demonstrated the inhibition effect of AHL lactonases on QS-regulated biofilm formation in *P. aeruginosa*.

In most instances, the transcribed levels of QS-coordinated receptor genes and virulence genes were significantly decreased by AiiA_S1-5_ at the late exponential phases of *A. hydrophila* and *P. aeruginosa* (Figure 5). However, the inhibitory effect was no longer observed at the stationary phase. This observation is consistent with that reported in an earlier study (Park et al., 2005; Sivakumar et al., 2019), and it was probably due to the accumulation of enzymatic inhibitors in the culture over time, or to a decrease in the metabolic activities of the cultures at the stationary phase. QQ enzymes should therefore be deployed as an environmentally benign approach for controlling microbe-associated problems at the early stages of growth and biofilm formation.

## Conclusion

In this study, bacterial species that proliferate under saline conditions were isolated and screened to select the ones that are positive for QQ under high salinity and high temperatures. Further genomic and biochemical characterization revealed a particularly promising AiiA from the *Altererythrobacter* sp. S1-5 that demonstrated a broad AHL substrate specificity range and enzymatic activity at an optimal pH of 8 and 50°C. AiiA_S1-5_ was also able to maintain good relative QQ activity with increasing salt concentrations up to 2 M. Its QQ efficacy against *P. aeruginosa, A. hydrophila*, and *V. alginolyticus* was further demonstrated, in that the motility traits and virulence gene cascades were detrimentally impacted, especially at the mid to late exponential phases of bacterial growth.

These findings collectively suggest that this QQ enzyme would be feasible for use in mitigating membrane biofouling under saline conditions and/or QS-associated pathogenic infections in marine aquaculture.

## Author Contributions

T-NW designed and performed the experiments, performed the data analysis, and wrote the manuscript with P-YH, who also supervised the research and provided reagents and materials. Q-TG and AP assisted with the genomic DNA sequence assembly. AK helped to write the manuscript.

## Conflict of Interest Statement

The authors declare that the research was conducted in the absence of any commercial or financial relationships that could be construed as a potential conflict of interest.
