# Supplementary material for: Discovering, Characterizing, and Applying Acyl Homoserine Lactone-Quenching Enzymes to Mitigate Microbe-Associated Problems Under Saline Conditions
Source: Front Microbiol. 2019 Apr 17;10:823. doi: 10.3389/fmicb.2019.00823 (PMC6479171; doi:10.3389/fmicb.2019.00823)
Supplement: Supplementary file 1 [file Data_Sheet_1.docx]

Supplementary Material

Discovering, Characterizing, and Applying Acyl Homoserine Lactone-Quenching Enzymes to Mitigate Microbe-Associated Problems Under Saline Conditions

Tian-Nyu Wang^1^, Qing-Tian Guan^2^, Arnab Pain^2^, Anna H. Kaksonen^3^, Pei-Ying Hong^1*^

^1^King Abdullah University of Science and Technology (KAUST), Water Desalination and Reuse Center (WDRC), Division of Biological and Environmental Science and Engineering (BESE), Thuwal, 23955-6900, Saudi Arabia

^2^ King Abdullah University of Science and Technology (KAUST), Pathogen Genomics Laboratory, Division of Biological and Environmental Science and Engineering (BESE), Thuwal, 23955-6900, Saudi Arabia

^3^ CSIRO Land and Water, 147 Underwood Avenue, Floreat WA 6014, Australia

*** Correspondence:**Dr. Pei-Ying Hong
[peiying.hong@kaust.edu.sa](mailto:peiying.hong@kaust.edu.sa)

Supplementary Table 1. Primers used in this study for AHL-quenching gene expression

| **Name** | **Sequence** | **Note** |
| --- | --- | --- |
| AiiA_S1-5_-F | CCGCTCGAGATGAAACGACTTCTGGCG | 5’ region flanked with *Xho*I restriction site |
| AiiA_S1-5_-R | TCGGAATTCTCAGCGGGCCGCCTCCGG | 5’ region flanked with *Eco*RI restriction site |
| SDR_S1-5_-F | TTAAGAAGGAGATATACATATGAGTCTCCTCAGCAGCC |  |
| SDR_S1-5_-R | CGACGGAGCTCGAATTCGGCCCTCGGCCGAGAAAGGGG |  |
| Est_S1-5_-F | CGCGGATCCATGATGCTTGCTCGTCTTGCCG | 5’ region flanked with *Bam*HI restriction site |
| Est_S1-5_-R | CCGGAATTCTCAGCGGCGATGACGCCAG | 5’ region flanked with *Eco*RI restriction site |
| Hyd_S1-5_-F | CGCGGATCCATGAAGACGCCGAAGTTTC | 5’ region flanked with *Bam*HI restriction site |
| Hyd_S1-5_-R | TTGGAATTCTCAGCGCGCGTCGGCGCG | 5’ region flanked with *Eco*RI restriction site |
| Hyd_L11_-F | GAAGGAGATATACATATGAAATTGAACTTTGAAATTAG |  |
| Hyd_L11_-R | CGACGGAGCTCGAATTCGGGGGGCGGTTTAAAAAATC |  |
| PvdQ_L11_-F | TTAAGAAGGAGATATACATATGTTAATTTGGGTAAAACG |  |
| PvdQ_L11_-R | CGACGGAGCTCGAATTCGGTAGCTTTGGCCTAAAGTC |  |
| SDR_L11_-F | TTAAGAAGGAGATATACATATGTCGAACAACATTCAAG |  |
| SDR_L11_-R | CGACGGAGCTCGAATTCGGAACTTCTTGTCGAGTTGG |  |
| HP_L11_-F | TTAAGAAGGAGATATACATATGAGTGACAAAGTTAAAATTG |  |
| HP_L11_-R | CGACGGAGCTCGAATTCGGGATTCCGTAACGTAATTTTAAG |  |

**Supplementary Table 2.** Selected precursor and product ion m/z values, retention times and mass spectrometer parameters used for Multiple Reaction Monitoring (MRM) analysis of 3-oxo-C12-HSL in LC-MS-MS experiment

| **Compound** | **3-oxo-C12-HSL** |
| --- | --- |
| w/m neutral Da | 297.3 |
| Retention time (min) | 14.2 |
| Precursor ion m/za | 298.155 |
| Product ion m/za | 102.000 & 197.000 |
| Entrance Potential (EP) | 10 |
| Declustering potential (DP) | 150 |
| Collision energy (CE) | 47 |
| Collision cell exit potential (CXP) | 14 |
| collision gas (CAD) | High |
| Ionspray voltage (IS) | 5500 |
| Temperature of ion source (TEM) | 0 |
| Nebulizing gas (GS1) | 13 |
| Drying gas (GS2) | 0 |
| Curtain Gas (CUR) | 20 |

**Supplementary Table 3.** Selected species used in this study to test the performance of AiiA_S1-5_ on marine strain biofilm control

| **Species** | **AHL** | **Quorum sensing regulated activity** | **Strains used in this study** | **Source** |
| --- | --- | --- | --- | --- |
| *Aeromonas hydrophila* | C4-HSL,C6-HSL, C5-HSL  (Talagrand-Reboul et al., 2017) | Biofilm formation, protease, production, virulence | *A. hydrophila* IN8 | Wastewater from KAUST (Al-Jassim et al., 2015) |
|  |  |  |  |  |
| *Pseudomonas aeruginosa* | C4-HSL, C6-HSL, 3-oxo-C6-HSL,3-oxo-C12-HSL  (Kusar et al., 2016) | Biofilm formation, virulence-related activity | *P. aeruginosa* DSM 1117 | DSMZ |
| *Vibrio alginolyticus* | C6-HSL, C8-HSL, 3-oxo-C6-HSL, 3-oxo-C12-HSL  (Liu et al., 2017) | extracellular toxin production, biofilm formation | *V. alginolyticus* V3 | Beach sand |

**Supplementary Table 4**. Information of virulence-associated genes for RT-qPCR study, amplification efficiency and regression coefficients of standard curves.

| **Strain** | **Gene** | **Annotation** | **Forward primer** | **Reverse primer** | **RT-qPCR amplification efficiency** | **RT-qPCR-based standard curve regression coefficient (R^2^)** | **Function** |
| --- | --- | --- | --- | --- | --- | --- | --- |
| *Aeromonas hydrophila* | ahyR | Transcriptional activator protein | TTCAACCAGTGCCCAGACTC | GCCCTCTTGCAGAAAACGC | 97.27 | 0.9987 | Quorum sensing dependent transcriptional regulator that mediate exoprotease production (Swift et al., 1999) |
|  | RpoB | DNA-directed RNA polymerase subunit beta | TTCATGGACCAGAACAACCC | GTCTCGAACTTCAAAGCCGG | 104.24 | 0.9966 | Reference gene |
| *Pseudomonas aeruginosa* | LasR | Transcriptional regulator | TCGAACATCCGGTCAGCAAA | CACCGAACTTCCGCCGAATA | 99.36 | 0.9993 | Global regulator of *P. aeruginosa* virulence genes including LasA/B, ToxA and AprA (Pearson et al., 1994). |
|  | AprA | [Alkaline metalloproteinase](https://www.ncbi.nlm.nih.gov/gene/881248) | ACGCCGTGGAAGTATGTCAG | GGATTGCAGCGACAACTTGG | 100.07 | 0.9996 | Extracellular virulence factors that mediate the pathogenic activity of *P. aeruginosa* (Pearson et al., 1994). |
|  | LasB | Elastase | TGTTCTATCCGCTGGTGTCG | GTTCATTCCGCCTGATTGCC | 95.40 | 0.9996 |  |
|  | ToxA | Exotoxin A | ATGCCACCTTCTTCGTCAGG | GCTGGGCGAGGTAGTTGTAG | 92.68 | 0.9994 |  |
|  | RpoB | DNA-directed RNA polymerase subunit beta | TTCCGAGATCACCCACAAGC | CCAGGGAGTTGATCAGACCG | 90.39 | 0.9983 | Reference gene |
| *Vibrio alginolyticus* | LuxR | LuxR family transcriptional regulator | GGTTCGAGTGGAGTGCTTCA | CGGTTCGCTTGGACAAACAG | 101.83 | 0.9997 | QS-mediated Regulator of virulence-related genes (Rui et al., 2008) |
|  | Pep | Peptidase | GTGGAAGGTTGCTGGGTCAA | CTTGAAGGGGATACTGGCGG | 107.08 | 0.9969 | QS-regulated Peptidase that involved in motility of *V. alginolyticus* (Cao et al., 2011) |
|  | RpoB | DNA-directed RNA polymerase subunit beta | TATCGGCCGTGAAGATGCTC | GGATACGACGGTTGCCTAGG | 95.44 | 0.9996 | Reference gene |

**Supplementary Table 5**. List of marine bacteria isolates used for AHL quenching activity test. AHL quenching level of each strain was classified into 5 levels based on their relative AHL quenching efficiency, A: 90-100%, B: 60-90%, C: 40-60%, D: 20-40%, E: 0-20%, -: not detected

| **Sampling place** | **Isolate** | **Closely related strain (Accession number)** | **Length**  **(bp)** | **Total score** | **E value** | **Identity** | **AHL quenching level** |
| --- | --- | --- | --- | --- | --- | --- | --- |
| **Marine aquaculture sludge** | L1 | *Staphylococcus aureus* (CP019594.2) | 1298 | 2375 | 0.0 | 99% | C |
|  | L2 | *Staphylococcus aureus* subsp. *aureus* (KF068119.1) | 1251 | 2300 | 0.0 | 99% | C |
|  | L3 | *Tamlana* sp. (KY436490.1) | 1212 | 2218 | 0.0 | 99% | C |
|  | L4 | *Tamlana crocina* (NR_042535) | 817 | 1431 | 0.0 | 99% | C |
|  | L5 | *Tenacibaculum discolor* (NR_042576) | 812 | 1443 | 0.0 | 99% | B |
|  | L6 | *Halobacillus* sp. (KF933643.1) | 1407 | 2593 | 0.0 | 99% | - |
|  | L7 | *Mesoflavibacter zeaxanthinifaciens* (NR_114033) | 760 | 1353 | 0.0 | 99% | B |
|  | L8 | *Halobacillus kuroshimensis* (KR347273.1) | 1419 | 2604 | 0.0 | 99% | B |
|  | L9 | *Virgibacillus dokdonensis* (NR_043206) | 858 | 1512 | 0.0 | 99% | C |
|  | L10 | *Pontibacillus chungwhensis* (NR_025812) | 909 | 1613 | 0.0 | 99% | D |
|  | L11 | *Pseudoalteromonas* sp. (JQ237129.1) | 1500 | 2521 | 0.0 | 98% | A |
|  | L12 | *Pontibacillus* sp*.* (MG252492.1) | 826 | 1487 | 0.0 | 99% | A |
|  | L14 | *Thalassobacillus hwangdonensis* (NR_104552) | 853 | 1519 | 0.0 | 99% | C |
|  | L15 | *Halobacillus trueperi* (NR_025459) | 860 | 1516 | 0.0 | 99% | E |
| **Beach sand** | T1 | *Bacillus* sp. (JQ946069.1) | 587 | 1048 | 0.0 | 99% | C |
|  | T2 | *Bacillus* sp. (GQ249102.1) | 1196 | 2141 | 0.0 | 99% | B |
|  | T3 | *Bacillus pumilus* (EU741079.1) | 1310 | 2343 | 0.0 | 99% | C |
|  | T5 | *Bacillus foraminis* (KC734537.1) | 1197 | 2120 | 0.0 | 99% | C |
|  | T7 | *Bacillus firmus* (KF601694.1) | 1175 | 2067 | 0.0 | 99% | B |
|  | T8 | *Bacillus jeotgali* (GU397390.1) | 1374 | 2446 | 0.0 | 99% | B |
|  | T9 | *Bacillus* sp. (AB698789.1) | 1144 | 2013 | 0.0 | 99% | C |
|  | T10 | *Delftia lacustris* (KF054933.1) | 1056 | 1884 | 0.0 | 99% | C |
|  | T12 | *Bacillus megaterium* (KC250230.1) | 1119 | 2010 | 0.0 | 99% | - |
|  | T13 | *Bacillus amyloliquefaciens* (HG514499.1) | 1148 | 2057 | 0.0 | 100% | C |
|  | T14 | *Bacillus megaterium* (KF475802.1) | 1368 | 2466 | 0.0 | 100% | - |
|  | T15 | *Bacillus* sp. (KC835068.1) | 1153 | 2042 | 0.0 | 99% | - |
|  | T17 | *Bacillus subtilis* (KC443104.1) | 1360 | 2453 | 0.0 | 100% | D |
|  | T18 | *Bacillus circulans* (HE575921.1) | 1308 | 2066 | 0.0 | 96% | C |
|  | T19 | *Bacillus subtilis* (HQ858061.1) | 1305 | 2343 | 0.0 | 99% | C |
|  | T23 | *Bacillus halosaccharovorans* (MH429923.1) | 652 | 1194 | 0.0 | 99% | B |
|  | T25 | *Bacillus* sp*.* (AB698789.1) | 1330 | 2340 | 0.0 | 99% | D |
|  | S1-1 | *Altererythrobacter marinus* (MF716636.1) | 945 | 1736 | 0.0 | 99% | A |
|  | S1-2 | *Devosia hwasunensis* (AM393883.1) | 651 | 1164 | 0.0 | 99% | E |
|  | S1-3 | *Oricola cellulosilytica* (KX809757.1) | 1017 | 1714 | 0.0 | 97% | - |
|  | S1-4 | *Halomonas* sp. (EU308349.1) | 1522 | 2573 | 0.0 | 99% | C |
|  | S1-5 | *Altererythrobacter sp.* (KC169804.1) | 1447 | 2368 | 0.0 | 98% | A |
|  | S1-6 | *Altererythrobacter marinus* (NR_116432.1) | 967 | 1755 | 0.0 | 99% | A |
|  | S2-1 | *Bacillus subtilis* (MH569338.1) | 1459 | 1825 | 0.0 | 99% | C |
|  | S2-2 | *Bacillus aquimaris* (HQ234271.1) | 1279 | 2257 | 0.0 | 99% | B |
|  | S2-3 | *Virgibacillus koreensis*(KC844773.1) | 539 | 891 | 0.0 | 99% | C |
|  | S2-4 | *Aquibacillus koreensis* (MG195136.1) | 1310 | 2194 | 0.0 | 97% | C |
|  | S2-5 | *Aquibacillus koreensis* (KY427829.1) | 618 | 1120 | 0.0 | 99% | C |
|  | S2-6 | *Bacillus* sp. (HQ397053.1) | 1439 | 1724 | 0.0 | 99% | C |
|  | S2-7 | *Bacillus mesophilus* (NR_149175.1) | 609 | 1086 | 0.0 | 99% | E |
| **Seawater (Red Sea)** | S3-1 | *Bacillus oryzaecorticis* (NR_133977.1) | 563 | 1016 | 0.0 | 99% | C |
|  | S3-3 | *Bacterioplanes sanyensis* (NR_126264.1) | 1378 | 2518 | 0.0 | 99% | C |
|  | S3-4 | *Vibrio harveyi*(HQ161746.1) | 1338 | 2420 | 0.0 | 99% | D |
|  | S3-5 | *Bacillus* sp. (GQ280078.1) | 824 | 1502 | 0.0 | 99% | D |
|  | S3-6 | *Bacillus altitudinis* (MG645242.1) | 868 | 1572 | 0.0 | 99% | D |
|  | S3-8 | *Vibrio harveyi*(MG819722.1) | 1292 | 2348 | 0.0 | 99% | D |
|  | S3-9 | *Vibrio sinaloensis* (MG833245.1) | 1318 | 2390 | 0.0 | 99% | C |

**Supplementary Table 6**. AHL specificity of four ORFs against different AHL. Specificity was evaluated by adding 50 uL crude enzyme with different AHLs at 37 °C for 1 h. Residual AHL was determined by the *A. tumefaciens* bioassay. The + means relative AHL quenching efficiency of the crude enzyme was ≥15%. The tested AHL concentration was selected based on the detection sensitivity of the *A. tumefaciens* biosensor to each type of AHL molecule.

| **AHL** | **Tested concentration** | **AiiA_S1-5_** | **SDR_S1-5_** | **Est_S1-5_** | **SDR_L11_** |
| --- | --- | --- | --- | --- | --- |
| C4-HSL | 60 μM | + | - | - | - |
| C6-HSL | 10 μM | + | - | - | - |
| C8-HSL | 8 μM | + | - | + | + |
| C10-HSL | 60 μM | + | - | + | - |
| C12-HSL | 60 μM | + | - | + | - |
| 3-oxo-C6-HSL | 8 μM | + | + | + | + |
| 3-oxo-C8-HSL | 10 nM | + | + | + | + |
| 3-oxo-C10-HSL | 6 μM | + | + | + | + |
| 3-oxo-C12-HSL | 10 μM | + | + | + | + |

**Supplementary Table 7**. Salt bridges predicted in AiiA_S1-5._ Prediction of salt bridges from AiiA_S1-5_ without signal sequence was done using ESBRI (<http://bioinformatica.isa.cnr.it/ESBRI/introduction.html>). NH in Arg, NZ in Lys or NE & ND in His implies the side-chain nitrogen atom of positive charged groups, OD in Asp or OE in Glu suggests the side-chain carboxyl oxygen atom of negative charged groups.

| Residue 1 | Residue 2 | Distance |
| --- | --- | --- |
| NH2 ARG A 9 | OD1 ASP A 11 | 3.57 |
| NZ LYS A 34 | OD2 ASP A 66 | 3.25 |
| NE2 HIS A 46 | OD2 ASP A 159 | 3.58 |
| NH1 ARG A 73 | OD2 ASP A 11 | 3.23 |
| NH2 ARG A 73 | OD2 ASP A 72 | 3.56 |
| NE2 HIS A 96 | OD1 ASP A 192 | 3.59 |
| NE2 HIS A 96 | OD2 ASP A 192 | 2.95 |
| ND1 HIS A 101 | OD1 ASP A 192 | 3.95 |
| NE2 HIS A 101 | OD1 ASP A 100 | 3.05 |
| NE2 HIS A 101 | OD2 ASP A 100 | 3.68 |
| NE2 HIS A 101 | OD1 ASP A 192 | 2.89 |
| NE2 HIS A 101 | OD2 ASP A 192 | 3.25 |
| NH1 ARG A 163 | OD1 ASP A 155 | 3.04 |
| NH1 ARG A 163 | OD2 ASP A 155 | 2.68 |
| NE2 HIS A 170 | OD2 ASP A 192 | 2.93 |
| NH1 ARG A 202 | OE1 GLU A 201 | 2.80 |
| NH2 ARG A 202 | OE1 GLU A 201 | 2.69 |
| NH2 ARG A 212 | OD2 ASP A 25 | 2.64 |
| NH1 ARG A 221 | OD1 ASP A 153 | 3.50 |
| NH1 ARG A 221 | OD2 ASP A 153 | 2.64 |
| NH2 ARG A 221 | OD1 ASP A 153 | 2.61 |
| NH2 ARG A 221 | OD2 ASP A 153 | 3.37 |
| NZ LYS A 223 | OE1 GLU A 203 | 2.71 |
| NE2 HIS A 237 | OD1 ASP A 100 | 2.79 |

**Supplementary Figure 1**. Normalized β-galactosidase activity of residual AHL after 40 h reaction with bacteria in artificial seawater. NC denotes the residual AHL activity of the negative control sample (AHL mixture dissolved in the same HSAS buffer) after 40 h reaction. The bacteria that showed relative AHL quenching efficiency ≥90% was highlighted in orange.

| A | B |
| --- | --- |
| C | D |
|  |  |

**Supplementary Figure 2.** AHL quenching activity of each fraction of AHL quenching bacteria at 50 °C. **(A)** AHL quenching activity in cell extract and heat-inactivated cell extract at 50 °C, **(B)** AHL quenching activity of >3 kDa and <3 kDa fractions of cell extract at 50 °C, **(C)** AHL quenching activity in supernatant and heat-inactivated supernatant at 50 °C, **(D)** AHL quenching activity of >3 kDa and <3 kDa fractions of supernatant at 50 °C. Each fraction was mixed with AHL mixture (25 mg/L, each of C4-HSL, C6-HSL, C8-HSL and 3-oxo-C12-HSL) and incubated at 50°C for 18 h for residual AHL determination using biosensor. Relative AHL quenching efficiency of each sample was calculated based on equation 1 stated in materials and methods. PBS buffer and AS were used in replacement of cell extract and supernatant to constitute negative control. Two biological replicates were performed, and results were expressed as mean ± standard error.

| A  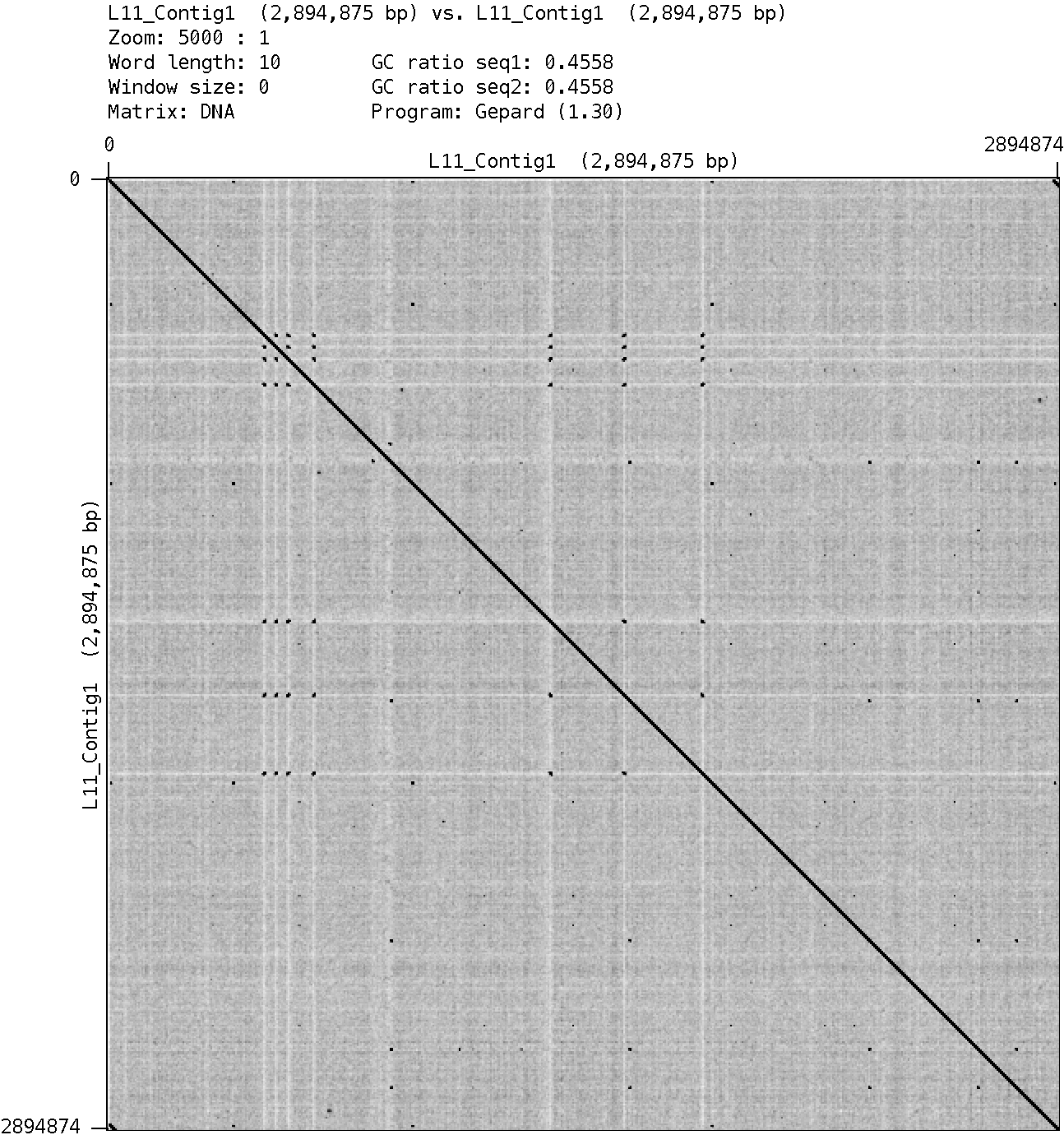 | B  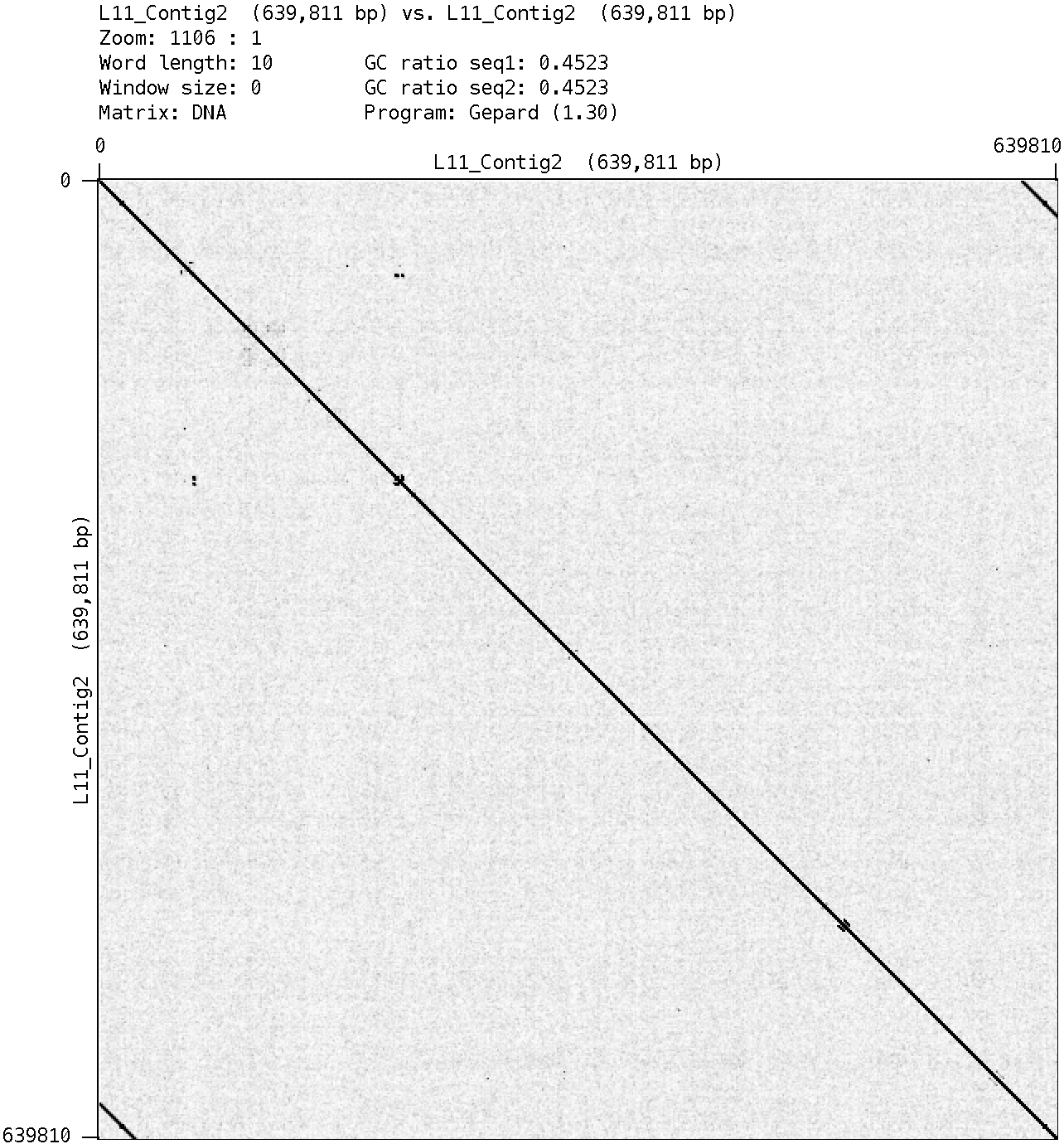 | C  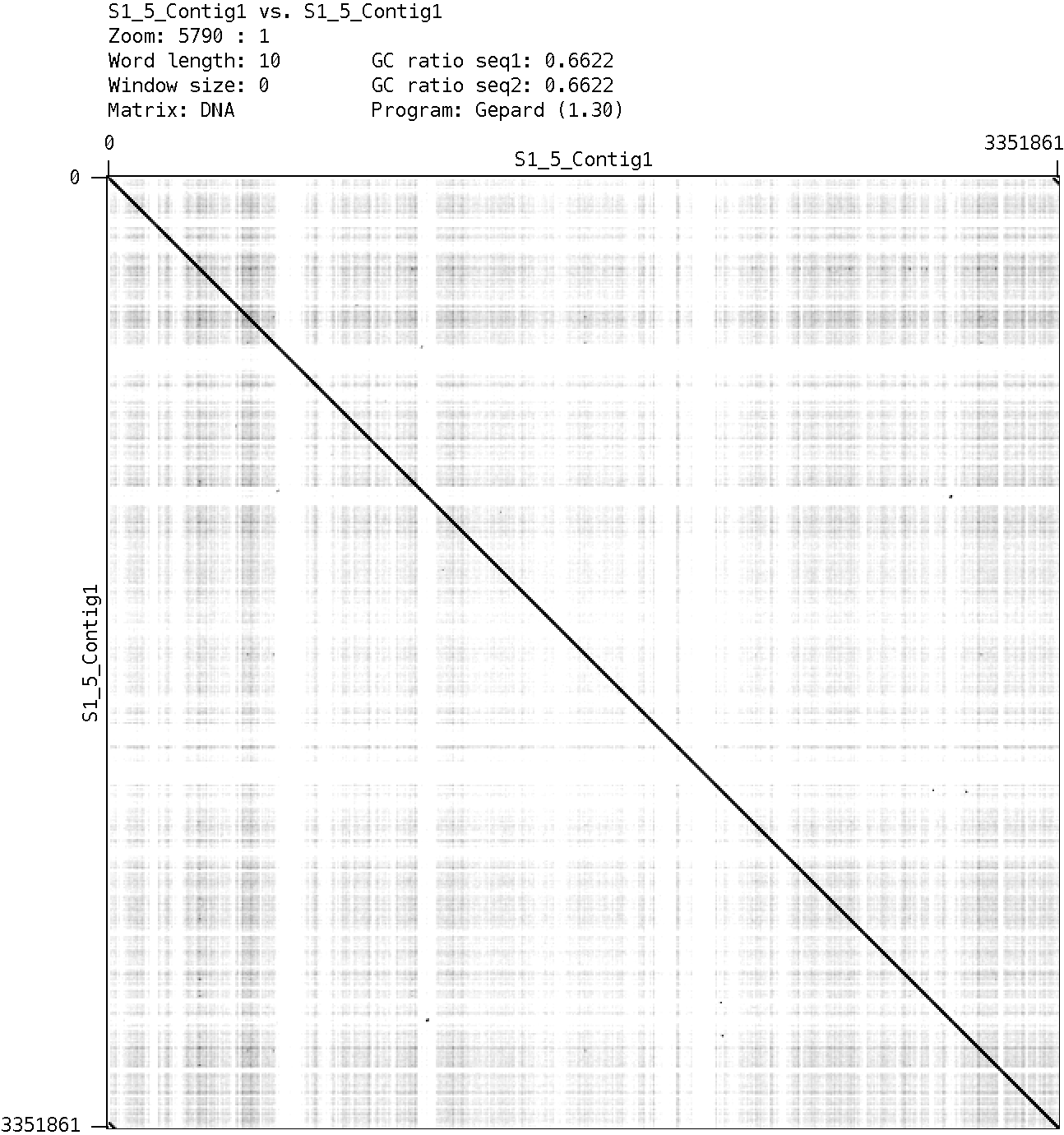 |
| --- | --- | --- |
|  |  |  |
| D  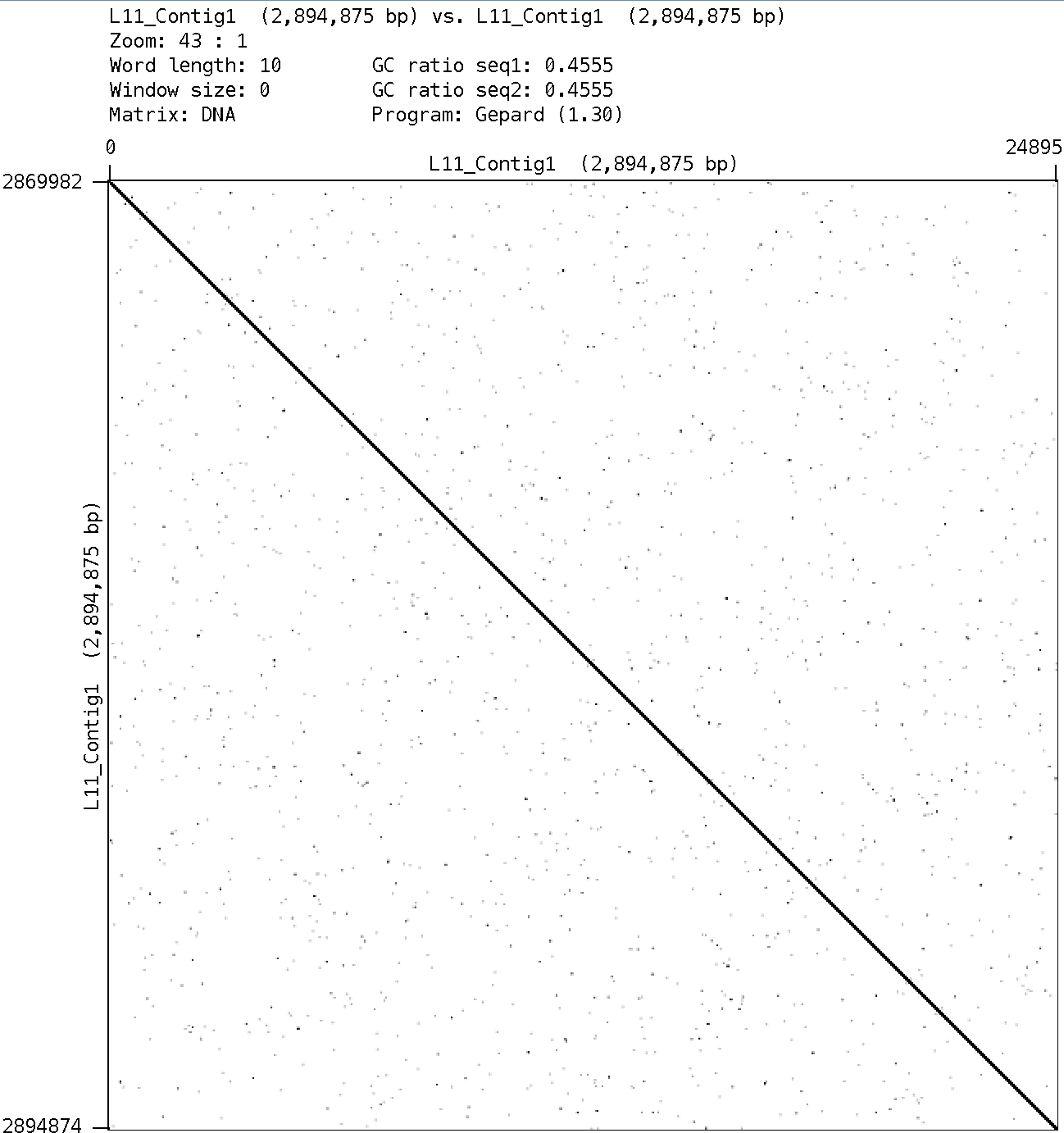 | E  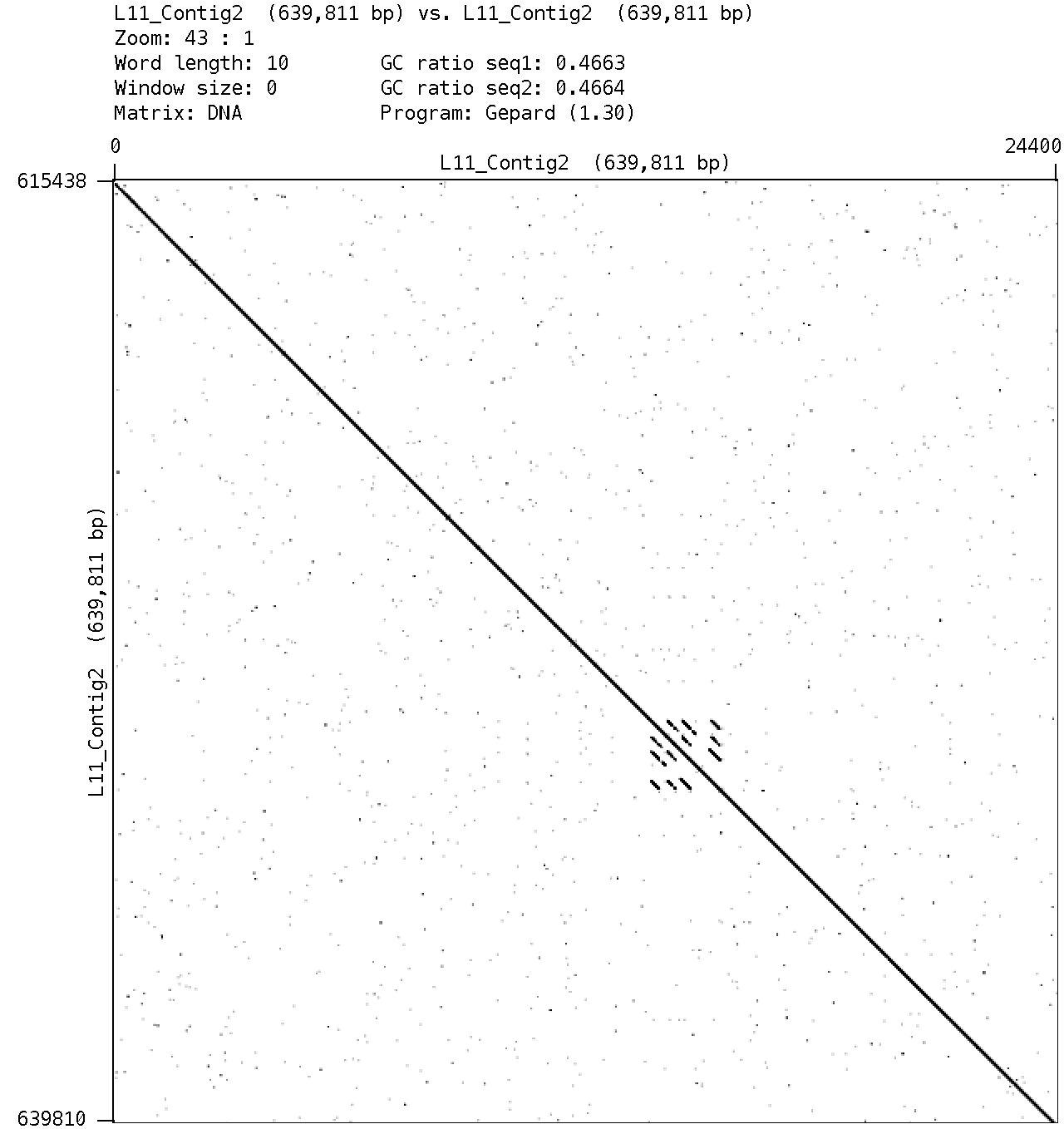 | F  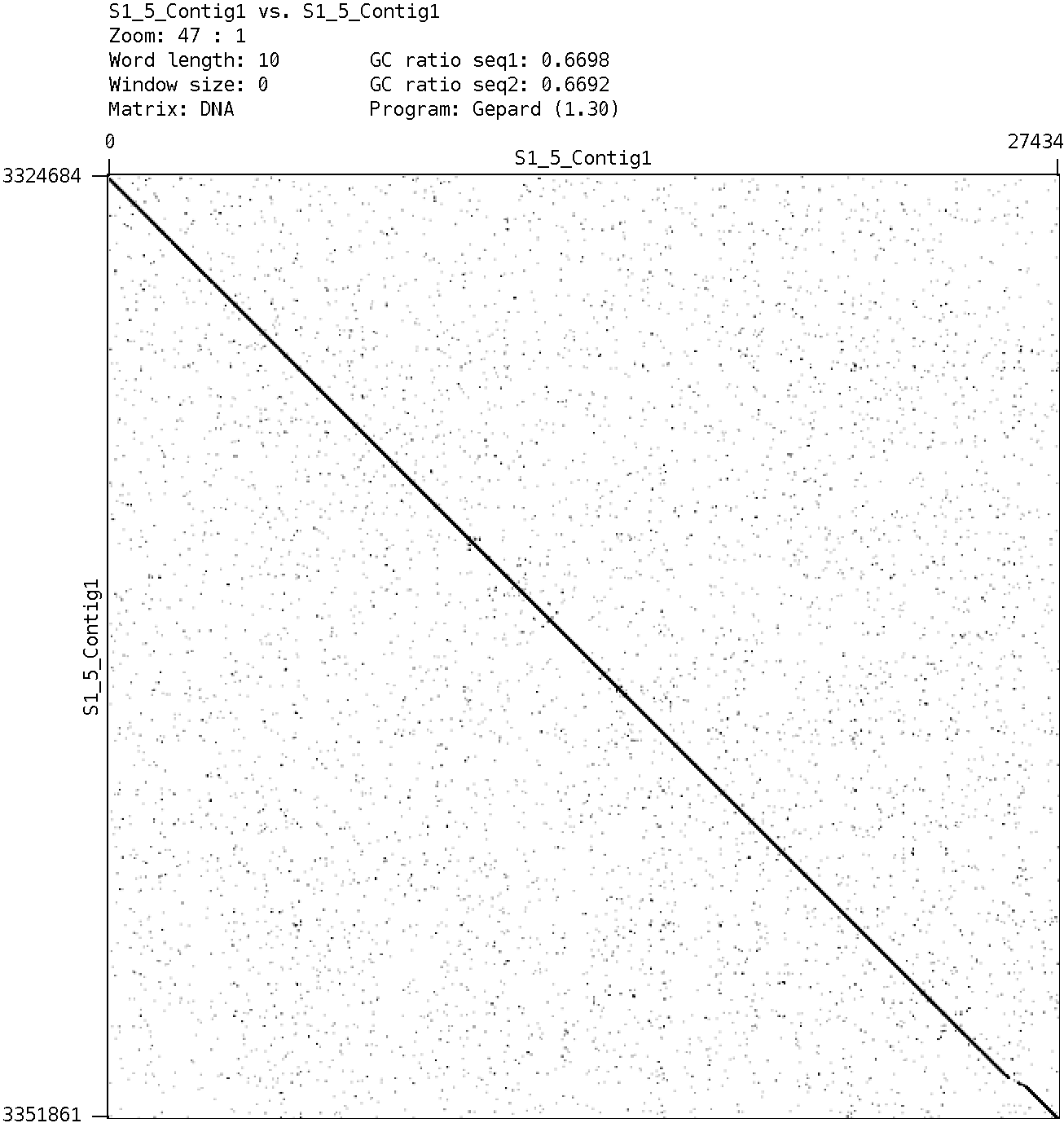 |

**Supplementary Figure 3.** The dot plot validation of assembled draft after contigs assembly. The dot plot of L11-contig 1 **(A)**, L11-contig 2 **(B)** and S1-5 contig **(C)** aligned to itselves shows a repetitive segments at the end of L11-contig1 from 2,869,982 bp to 2,894,874 bp **(D)**, from 615,438 bp to 639,810 bp in L11-contig 2 **(E)**, and from 3,324,684 bp to 3,351,861 bp in S1-5 contig **(F)**.

| A  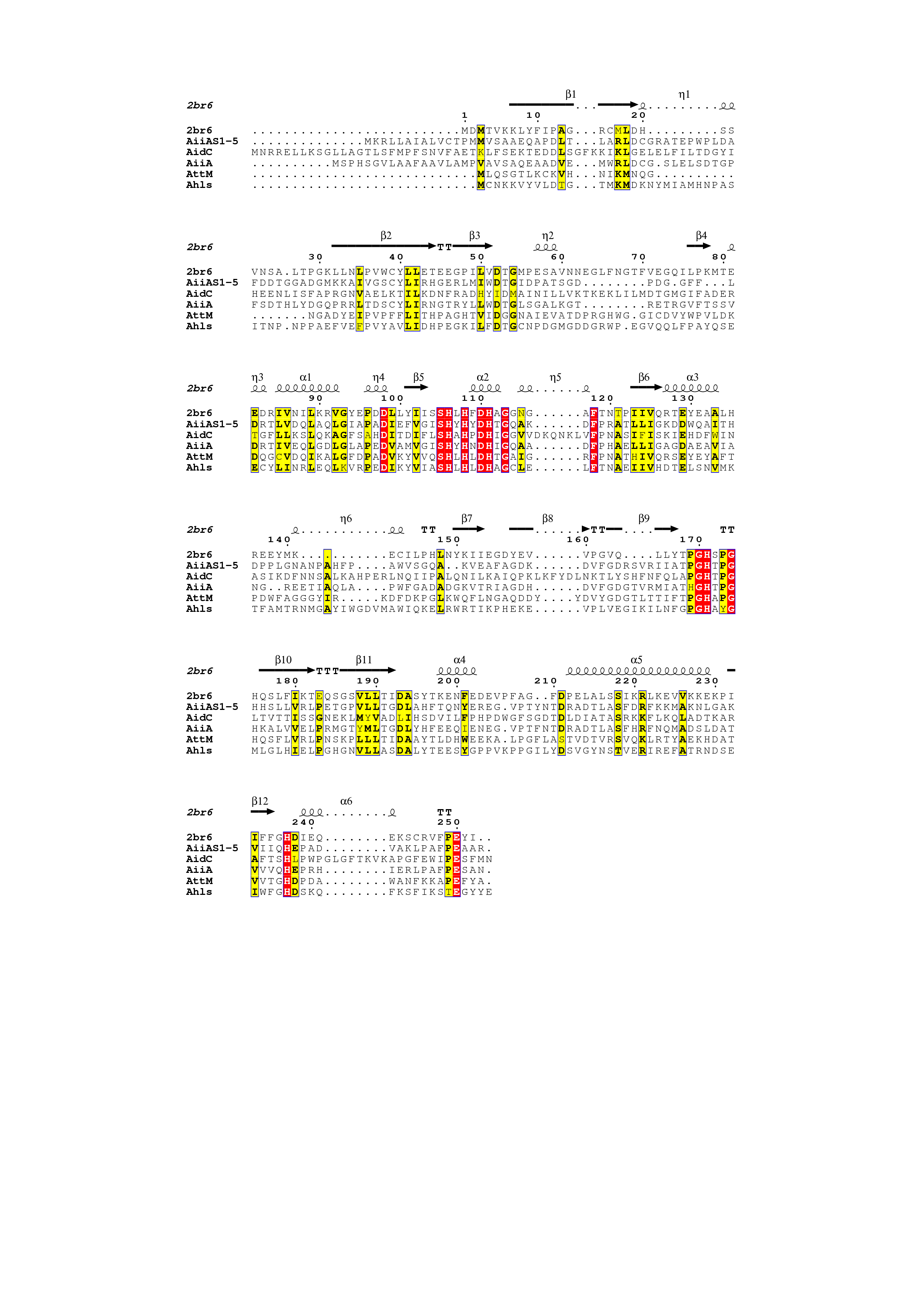 | B  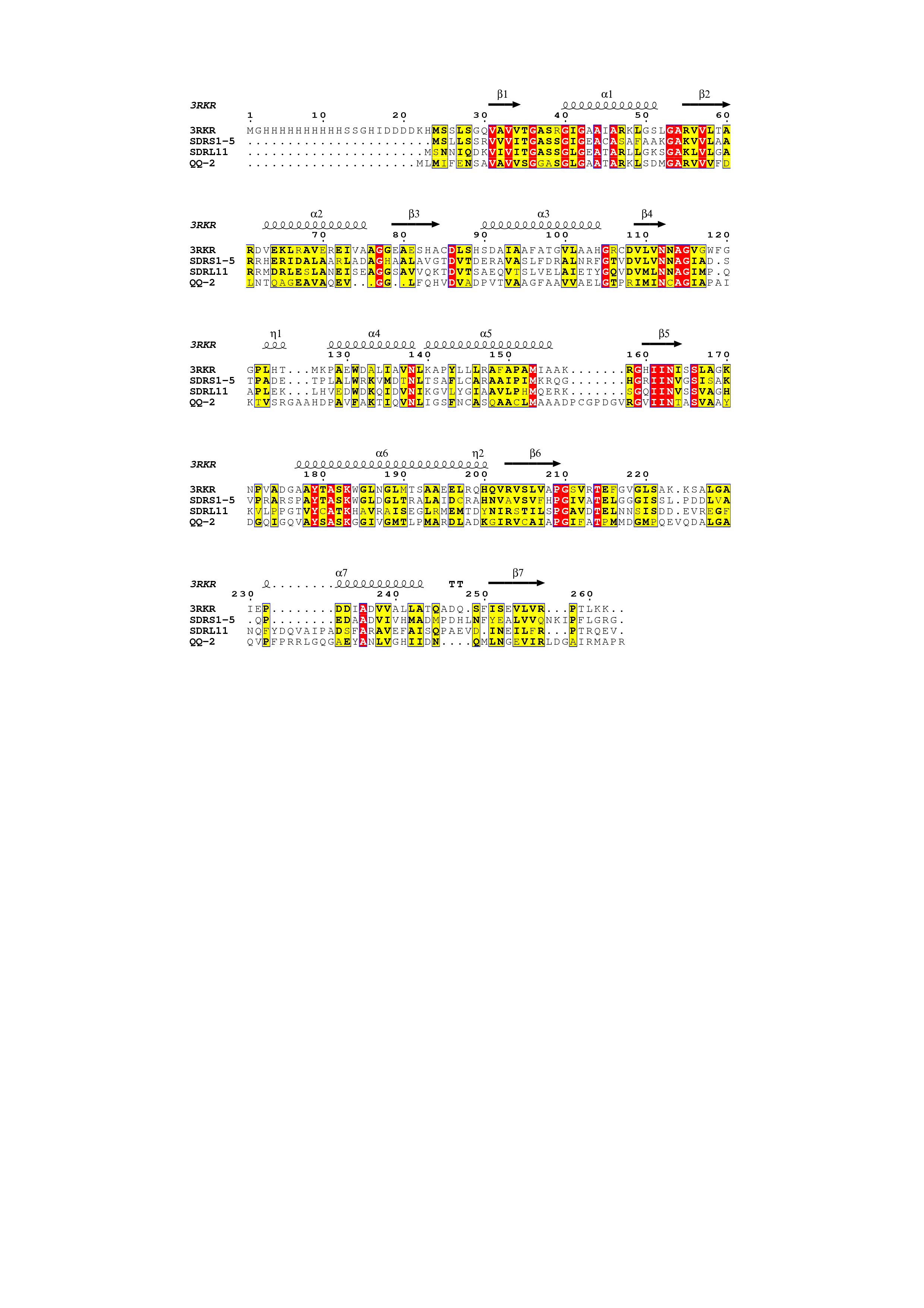 |
| --- | --- |
| C  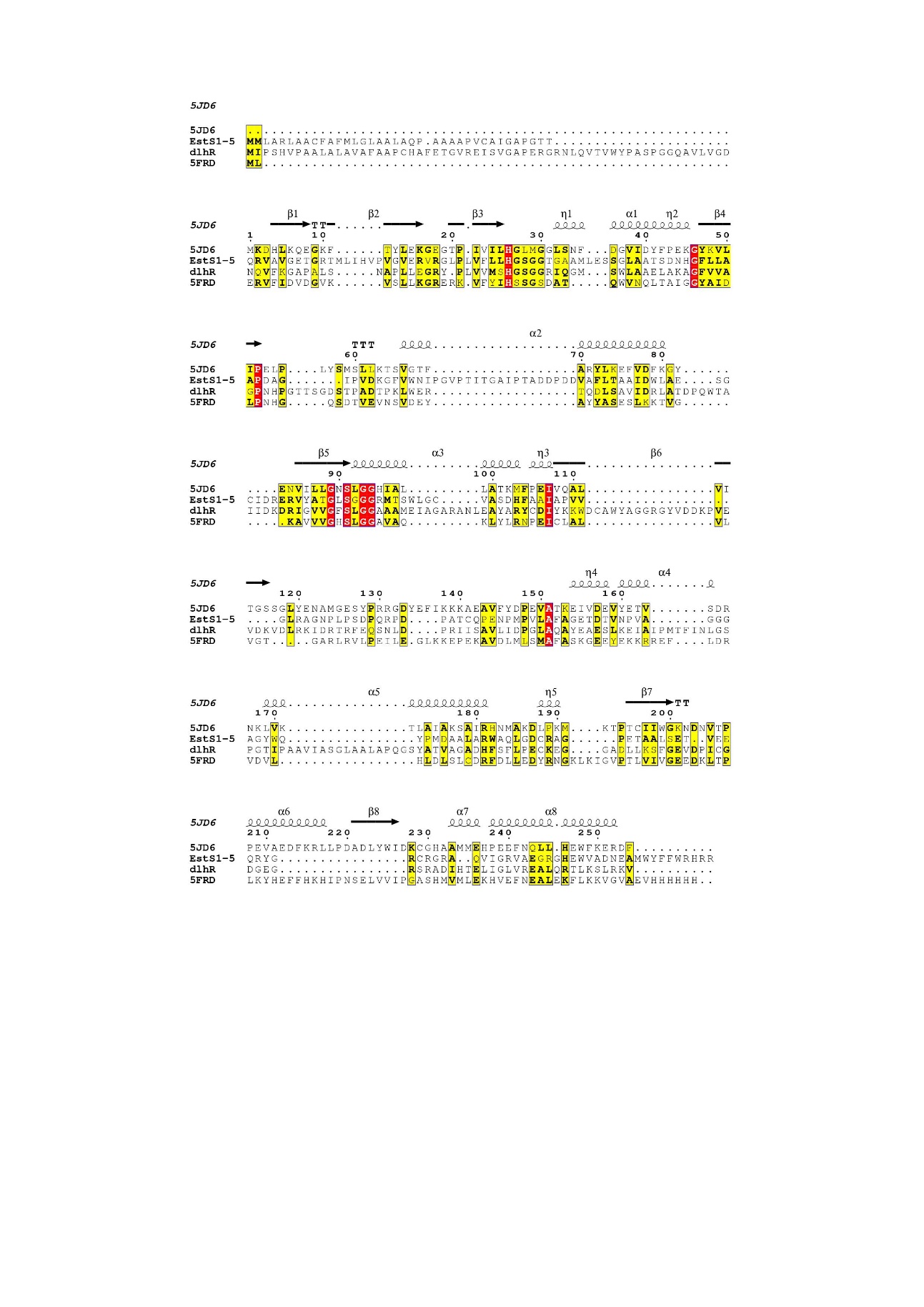 | D  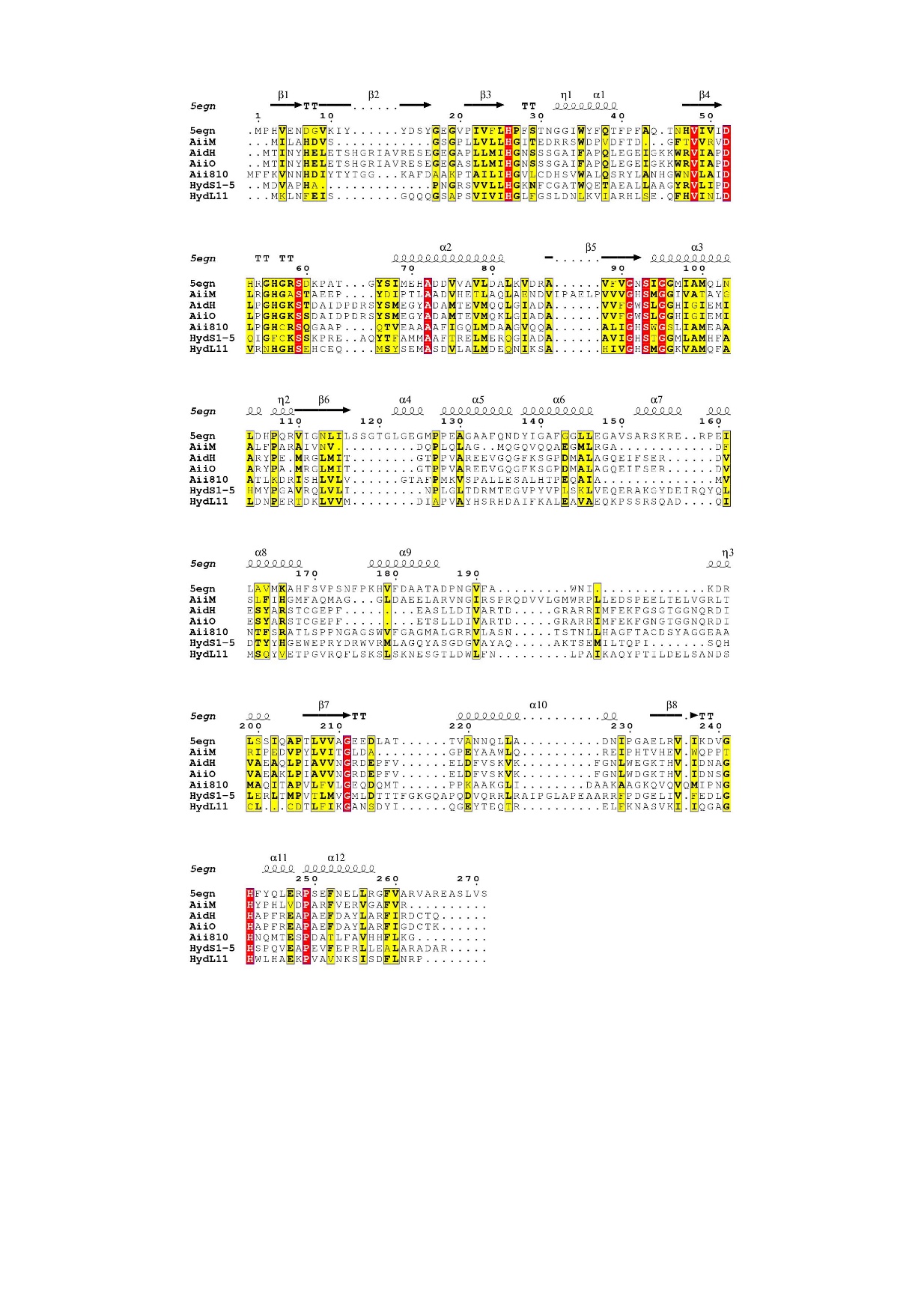 |
| E  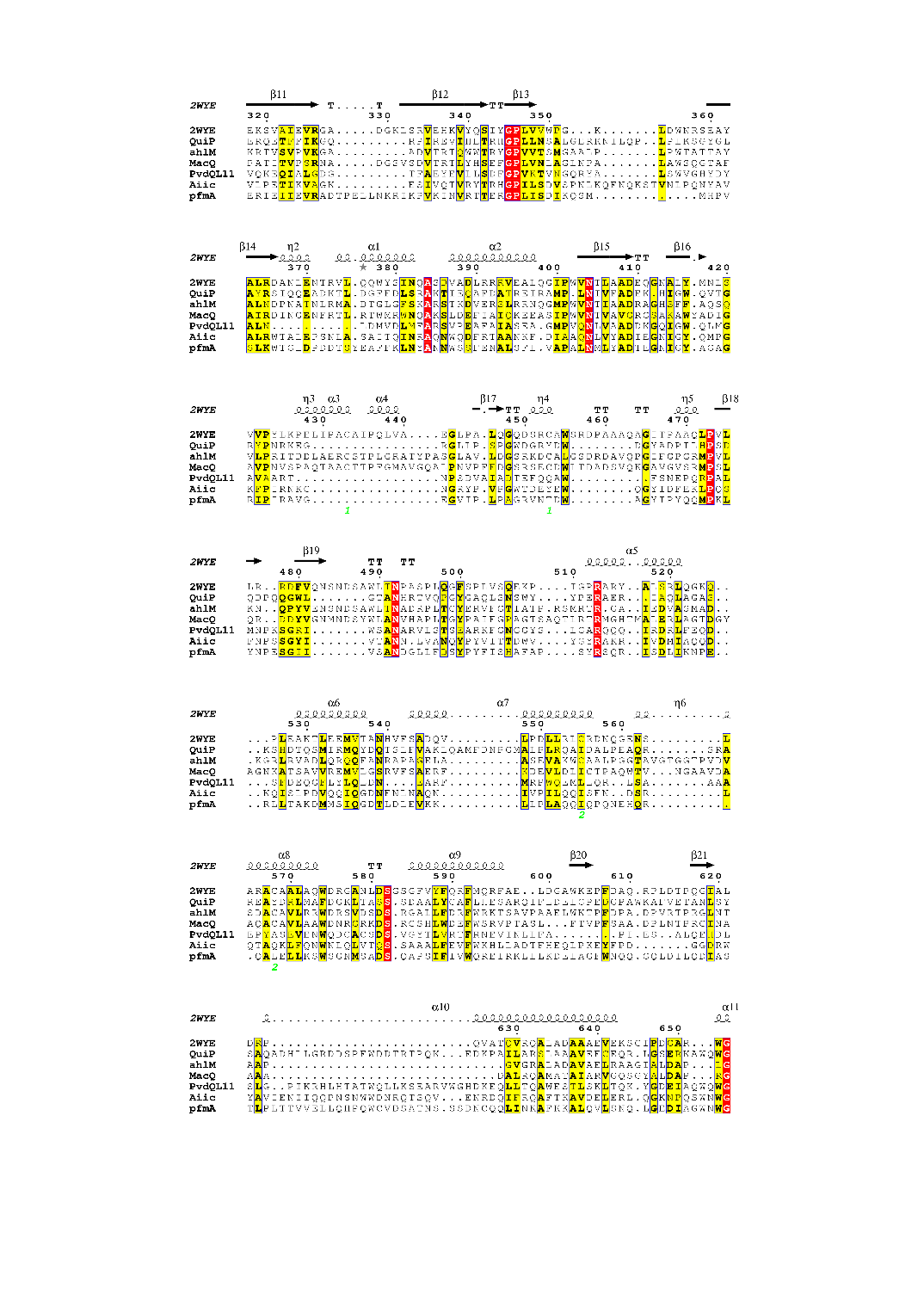 |  |

**Supplementary Figure 4**. Structure-based multiple sequence alignment of 8 ORFs and other reported AHL QQ enzymes. Sequence alignment was performed by the MUSCLE program in the MEGA 7.0 and expressed by ESPript 3.0. The secondary structure of the QQ protein is shown on top. α: α-helix; β: β-sheet; η: 3_10_-helice; T: β-turns/coils.  Red box with white character denotes conserved identity, the motif for AHL degradation was marked with a blue box.

**(A)** AiiAS1-5 multiple sequence alignment, 2BR6: AiiA from *Bacillus thuringiensis* subsp. kurstaki (2BR6_A), AiiA_S1-5_: AiiA from *Altererythrobacter* sp. S1-5, AidC: AiiA from *Chryseobacterium* sp. StRB126 (WP_045498117.1), AiiA: AiiA from *Erythrobacter flavus* VG1 (WP_067675887.1), AttM: AiiA from *Agrobacterium tumefaciens* C58 (WP_010974402.1), Ahls: AiiA from *Solibacillus silvestris* StLB046 (WP_065216334.1).

**(B)** SDR_S1-5_ and SDR_L11_ multiple sequence alignment, 3RKR: BpiB09 (SDR) from soil metagenome (3RKR_A), SDR_S1-5_: SDR from *Altererythrobacter* sp. S1-5, SDR_L11_: SDR from *Pseudoalteromonas* sp. L11, QQ2: SDR from Salt Marsh metagenome (AGH24763.1).

**(C)** Est_S1-5_ multiple sequence alignment, 5jd6: α/β hydrolase enzyme from metagenome of Sediments (5JD6_A), Est_S1-5_: esterase from *Altererythrobacter* sp. S1-5, dlhR: dienelactone hydrolase from *Sinorhizobium fredii* NGR234 (YP_002824413.1), 5frd: esterase from *Archaeoglobus fulgidus* (5FRD)

**(D)** HydS1-5 and HydL11 multiple sequence alignment, 5egn: Est816 from metagenome of Turban Basin (5EGN), AiiM: α/β hydrolase from *Microbacterium testaceum* StLB106 (BAK74766.1), AidH: α/β hydrolase from *Ochrobactrum* sp. T63 (ACZ73823.1), AiiO: α/β hydrolase from *Ochrobactrum* sp. A44 (WP_095447712.1), Aii810: α/β hydrolase from Mao-tofu metagenome (ASY06633.1)

**(E)** Partial result of Qvd_L11_ multiple sequence alignment, 2WYE: AHL Acylase PvdQ from *Pseudomonas aeruginosa* PAO1 (2WYE_B). QuiP second AHL acylase from *Pseudomonas Aeruginosa* PAO1 (WP_003112526.1), ahlM: AHL acylase from *Streptomyces* sp. strain M664 (AAT68473.1), MacQ: Penicillin and AHL acylase from *Acidovorax* sp. MR-S7 (WP_020227037.1), PvdQL11: AHL acylase from *Pseudoalteromonas* sp. L11, AiiC: AHL acylase (all3924) from *Nostoc* sp. PCC 7120 (BAB75623.1), pfmA: AHL acylase from *Pseudoalteromonas flavipulchra* JG1 (ASS36259.1)

| A | B |
| --- | --- |

**Supplementary Figure 5.** Relative enzyme activity of **(A)** AiiA_S1-5_ and **(B)** Est_S1-5_ in different concentration of KCl (0, 0.1, 0.5, 1 and 2 M). Relative enzyme activity of purified enzyme was normalized by the activity of purified enzyme at optimal saline condition. Reaction was performed in triplicates and error bars represents the standard deviation for the data points.

| **Strain name** | **Swarming** | | **Swimming** | |
| --- | --- | --- | --- | --- |
|  | **PBS buffer** | **AiiA_S1-5_** | **PBS buffer** | **AiiA_S1-5_** |
| *A. hydrophila* | 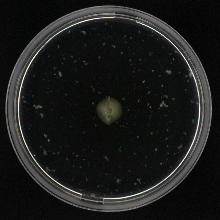 | 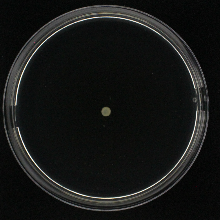 | 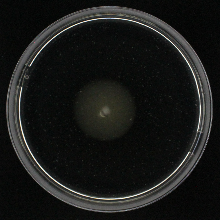 | 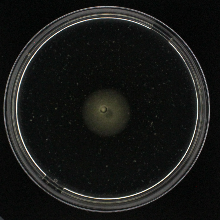 |
| *P. aeruginosa* | 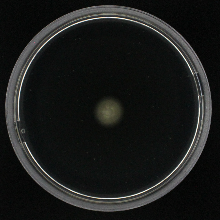 | 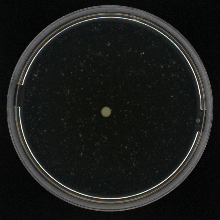 | 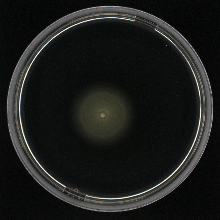 | 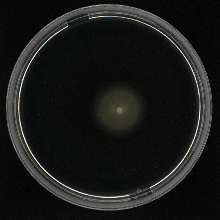 |
| *V. alginolyticus* | 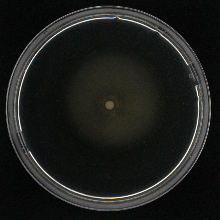 | 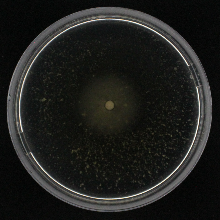 | 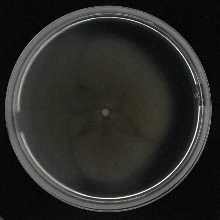 | 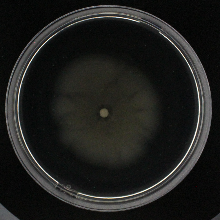 |

**Supplementary Figure 6**. Effect of purified AiiA_S1-5_ on motility of 3 opportunistic pathogens grown in saline conditions.

| A  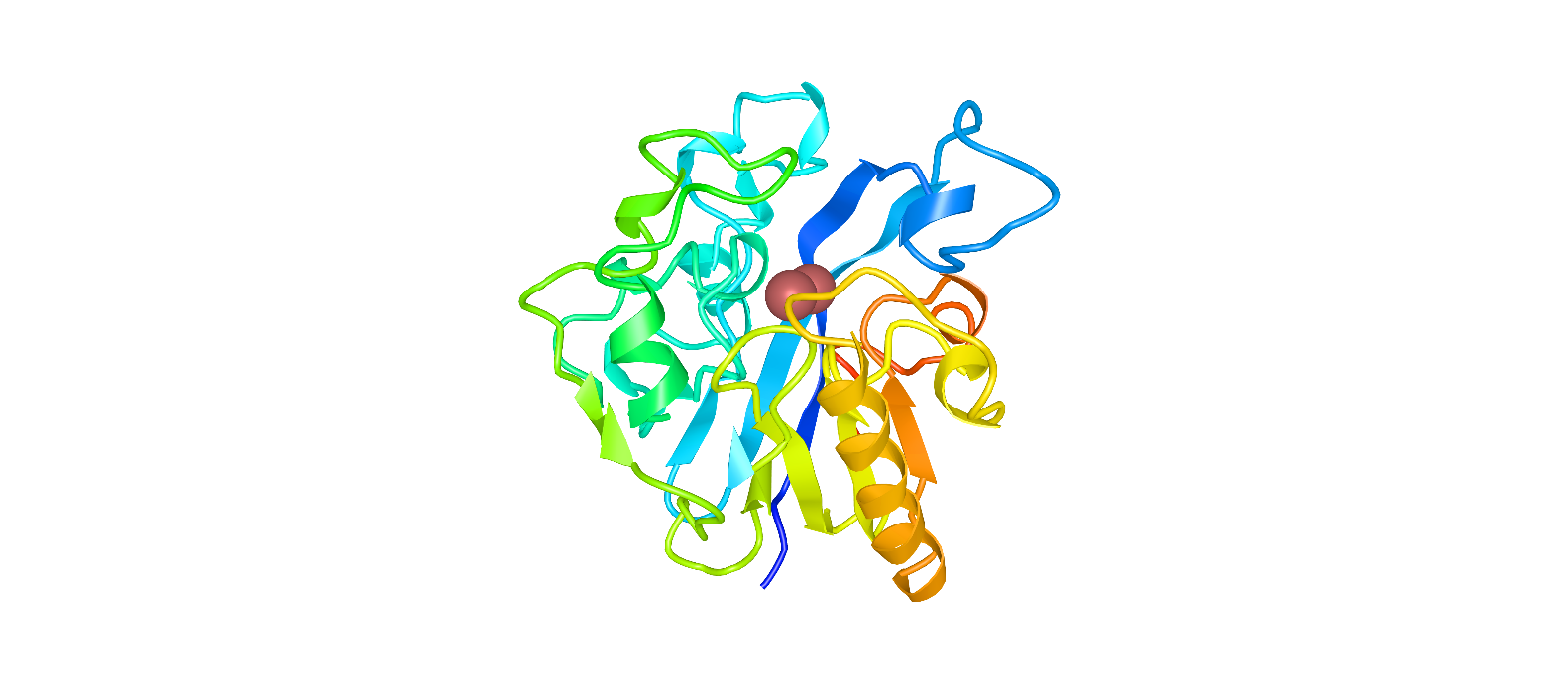 | B  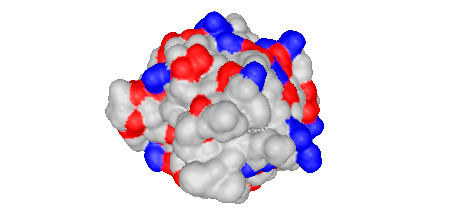 | C  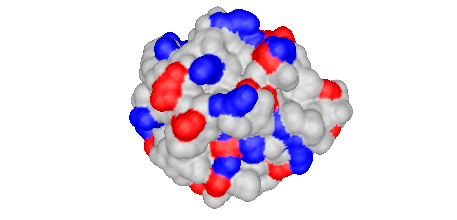 |
| --- | --- | --- |
|  | D  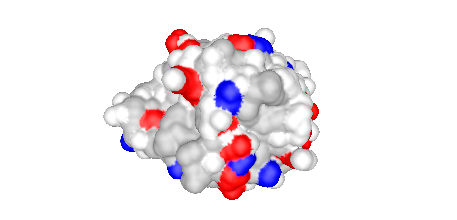 | E  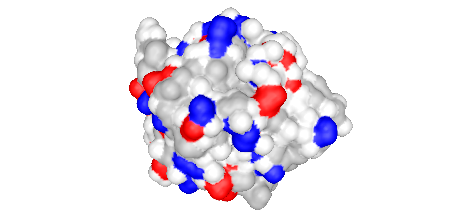 |

**Supplementary Figure 7.** Distribution of surface charge in AiiA_S1-5_ and reference model AiiA. **(A)** Putative tertiary structure of AiiA_S1-5._ 3D structure of AiiA_S1-5_ without signal sequence was predicted with Swiss model (<https://swissmodel.expasy.org/>) using 2br6 as template, 2br6: AiiA from *Bacillus thuringiensis* subsp. kurstaki (2BR6_A), the copper ball represent Zinc ions. Back **(B)** and front **(C)** image of positive charges (blue) and negative charges (red) on solvent-assessable surface of AiiA_S1-5,_ Back **(D)** and front **(E)** image of positive charges and negative charges on solvent-assessable surface of template AiiA (2BR6_A). All the image was generated from NCBI's web-based 3D structure viewer (<https://www.ncbi.nlm.nih.gov/Structure/icn3d/full.html>).

**References**

Al-Jassim, N., Ansari, M.I., Harb, M., and Hong, P.Y. (2015). Removal of bacterial contaminants and antibiotic resistance genes by conventional wastewater treatment processes in Saudi Arabia: Is the treated wastewater safe to reuse for agricultural irrigation? *Water Research* 73**,** 277-290. doi: 10.1016/j.watres.2015.01.036.

Cao, X., Wang, Q., Liu, Q., Rui, H., Liu, H., and Zhang, Y. (2011). Identification of a luxO-regulated extracellular protein Pep and its roles in motility in *Vibrio alginolyticus*. *Microb Pathog.* 50(2)**,** 123-131. doi: 10.1016/j.micpath.2010.12.003.

Kusar, D., Srimpf, K., Isakovic, P., Kalsek, L., Hosseini, J., Zdovc, I., et al. (2016). Determination of N-acylhomoserine lactones of *Pseudomonas aeruginosa* in clinical samples from dogs with otitis externa. *BMC Vet Res.* 12(1)**,** 233. doi: 10.1186/s12917-016-0843-0.

Liu, J., Fu, K., Wang, Y., Wu, C., Li, F., Shi, L., et al. (2017). Detection of Diverse N-Acyl-Homoserine Lactones in *Vibrio alginolyticus* and Regulation of Biofilm Formation by N-(3-Oxodecanoyl) Homoserine Lactone In vitro. *Front Microbiol.* 8**,** 1097. doi: 10.3389/fmicb.2017.01097.

Pearson, J.P., Gray, K.M., Passador, L., Tucker, K.D., Eberhard, A., Iglewski, B.H., et al. (1994). Structure of the autoinducer required for expression of *Pseudomonas aeruginosa* virulence genes. *Proc Natl Acad Sci U S A.* 91(1)**,** 197-201.

Rui, H., Liu, Q., Ma, Y., Wang, Q., and Zhang, Y. (2008). Roles of LuxR in regulating extracellular alkaline serine protease A, extracellular polysaccharide and mobility of *Vibrio alginolyticus*. *FEMS Microbiol Lett.* 285(2)**,** 155-162. doi: 10.1111/j.1574-6968.2008.01185.x.

Swift, S., Lynch, M.J., Fish, L., Kirke, D.F., Tomas, J.M., Stewart, G.S., et al. (1999). Quorum sensing-dependent regulation and blockade of exoprotease production in *Aeromonas hydrophila*. *Infect Immun.* 67(10)**,** 5192-5199.

Talagrand-Reboul, E., Jumas-Bilak, E., and Lamy, B. (2017). The Social Life of *Aeromonas* through Biofilm and Quorum Sensing Systems. *Front Microbiol.* 8**,** 37. doi: 10.3389/fmicb.2017.00037.
